# Supplementary material for: Spacing effect improves generalization in biological and artificial systems
Source: Patterns (N Y). 2026 May 19;7(6):101564. doi: 10.1016/j.patter.2026.101564 (PMC13280723; doi:10.1016/j.patter.2026.101564)
Supplement: Document S2. Article plus supplemental information [file mmc2.pdf]

Article

Patterns

Spacing effect improves generalization in biological and artificial systems

Highlights

- Spaced temporal ensembling improves generalization in artificial neural networks
- Generalization peaks at intermediate spaced intervals and variation strength
- Periodic spacing outperforms shuffled schedules and raises the performance ceiling
- Spaced training and sensory variation jointly improve generalization in *Drosophila*

Authors

Guanglong Sun, Ning Huang, Hongwei Yan, ..., Bo Lei, Yi Zhong, Liyuan Wang

Correspondence

liyuanwang@tsinghua.edu.cn

In brief

The timing of practice shapes how well learning generalizes. This study links the spacing effect to spaced temporal ensembling, showing that structured variation over spaced intervals improves generalization in artificial neural networks and in *Drosophila* memory.

Sun et al., 2026, Patterns 7, 101564
June 12, 2026 © 2026 The Author(s). Published by Elsevier Inc.
https://doi.org/10.1016/j.patter.2026.101564

CellPress

Patterns

CellPress
OPEN ACCESS

Article

Spacing effect improves generalization in biological and artificial systems

Guanglong Sun,[1,2,5,6] Ning Huang,[1,2,6] Hongwei Yan,[1,2,5] Jun Zhou,[1,2] Qian Li,[4] Bo Lei,[5] Yi Zhong,[1,2] and Liyuan Wang[3,7,*]
[1]School of Life Sciences, IDG/McGovern Institute for Brain Research, Tsinghua University, Beijing, China
[2]Tsinghua-Peking Center for Life Sciences, Beijing, China
[3]Department of Psychological and Cognitive Sciences, Tsinghua University, Beijing, China
[4]School of Medicine, Shenzhen Campus of Sun Yat-Sen University, Shenzhen, Guangdong, China
[5]Beijing Academy of Artificial Intelligence, Beijing, China
[6]These authors contributed equally
[7]Lead contact
*Correspondence: liyuanwang@tsinghua.edu.cn
https://doi.org/10.1016/j.patter.2026.101564

THE BIGGER PICTURE Spacing practice over time, rather than repeating it back-to-back, reliably improves learning in many animals, including humans. This spacing effect is widely used in education and training, yet the reason it works so well and how to translate it into better artificial intelligence are still open questions. Modern artificial neural networks often achieve impressive accuracy but can generalize poorly when conditions shift, data are limited, or training signals are noisy. Understanding what spacing contributes to biological learning can therefore inspire simple, broadly applicable ways to make machine learning systems more reliable.

This work shows that a shared principle can connect biological learning and machine learning: generalization improves when experience is revisited across spaced intervals while introducing an appropriate amount of variation. In artificial neural networks, the study unifies widely used temporal ensemble strategies under a single framework and finds that both the spaced interval and the variation strength jointly shape training-to-test generalization, with best results at intermediate values. Additional controls show that periodic spacing is more effective than irregular schedules, that combining spacing at neuronal, synaptic, and network levels yields cumulative gains, and that spaced training tends to raise the achievable performance ceiling and improve robustness to parameter perturbations. In *Drosophila* olfactory conditioning, both longer inter-trial intervals and controlled sensory variation enhance memory generalization to related odors, supporting a convergent computational principle across systems. Looking ahead, spacing-inspired schedules may help improve robustness in applications where data distributions drift over time, such as health monitoring, robotics, and scientific sensing, and they motivate new experiments to uncover how neural circuits integrate variability across time to build flexible memories.

SUMMARY

Generalization is a fundamental criterion for evaluating learning effectiveness, a domain where biological intelligence excels yet artificial intelligence faces challenges. In biological learning and memory, the well-documented spacing effect shows that appropriately spaced intervals between learning trials significantly improve behavioral performance. While multiple theories have been proposed to explain its underlying mechanisms, one compelling hypothesis is that spaced training promotes integration of input and innate variations, thereby enhancing generalization to novel but related scenarios. Here, we examine this hypothesis by introducing a bio-inspired spacing effect into artificial neural networks, integrating input and innate variations across spaced intervals at neuronal, synaptic, and network levels. These spaced ensemble strategies yield significant performance gains across benchmark datasets and network architectures. Biological experiments on *Drosophila* further validate the complementary effect of appropriate variations and spaced intervals in improving generalization, which together reveal a convergent computational principle of biological learning and machine learning.

© 2026 The Author(s). Published by Elsevier Inc.
This is an open access article under the CC BY license (http://creativecommons.org/licenses/by/4.0/).

INTRODUCTION

The ability to generalize previously learned knowledge to novel but related scenarios is a hallmark of intelligent behavior. For example, a person who has learned to recognize another's voice can identify it again despite variations in background noise, pitch, or speaking speed. This underlies adaptive decision-making in everyday life and represents an almost innate advantage of biological intelligence (BI). In comparison, generalization from training data to similar test data is a fundamental objective of artificial intelligence (AI), especially for modern artificial neural network (ANN)-based systems.[1,2] Understanding the mechanisms that support robust generalization in BI offers a promising path toward improving generalization in AI. Conversely, implementing bio-inspired generalization strategies in ANNs allows for reverse engineering and computational validation of candidate biological hypotheses. This interdisciplinary research paradigm, known as NeuroAI,[1–4] holds promise for uncovering their convergent computational principles.

One well-documented phenomenon in biological learning and memory is the spacing effect[5]: spaced training with scattered repetitions over a certain period of time is significantly more effective than massed training with focused repetitions. This effect has been reported across species, from invertebrates to humans,[6–13] and across a wide range of representative learning tasks.[14–19] Several cognitive theories have been proposed to account for this effect.[20] The deficient-processing theory[7,21–23] suggests that massed repetitions allow insufficient time for essential biological processes such as protein synthesis and synaptic plasticity, thereby impairing knowledge encoding. The study-phase retrieval theory[24,25] posits that spaced trials facilitate the retrieval and strengthening of earlier memory traces, thereby enhancing knowledge retention. The encoding variability theory[26–29] suggests that spacing can introduce natural variations in inputs or internal states, producing a more diverse set of memory traces. Importantly, these accounts are not mutually exclusive and may be complementary. Among them, encoding variability emphasizes how structured variations accumulated across repetitions can support robust responses beyond the specific training instance, providing a comparably explicit handle on generalization.

A parallel mechanism for improving generalization has emerged in machine learning, known as ensemble learning (EL),[2,4,30–32] which aggregates multiple models that capture diverse aspects of the data distribution. Similar to the temporally evolving nature of biological learning, temporal ensemble strategies[33,34] leverage variability accumulated across training steps to construct more robust and generalizable representations (Figures 1A and 1B), which include three representative implementations: (1) dropout[35] introduces stochasticity at the neuronal level by randomly deactivating units during training, promoting the formation of diverse activation patterns; (2) weight averaging (WA),[36] often implemented via exponential moving average (EMA),[37] aggregates parameter snapshots throughout network training, which captures variability at the synaptic level; and (3) knowledge distillation (KD),[38] especially online KD[39] and self-KD,[40] transfers soft output distributions from temporally more advanced teacher models to student models, integrating variability at the network level.

Based on a unified formulation of temporal ensemble strategies, we revisit and computationally validate the encoding variability theory that may underlie the spacing effect in biological systems. Specifically, we incorporate bio-inspired spacing effects into ANNs at the neuronal, synaptic, and network levels (corresponding to dropout,[35] WA,[36] and KD,[38] respectively) by introducing controllable input and innate variations across spaced training intervals. Across a variety of benchmark datasets and network architectures, we observe a consistent inverted U-shaped relationship between training-to-test generalization and both the magnitude of variations and the duration of spaced intervals. Strikingly, either appropriate variation strength under a fixed spaced interval or appropriate spaced interval under fixed variation strength leads to considerable improvements in test performance. These findings point to a critical balance between structured variability and temporal spacing for enhanced generalization.

Guided by these computational insights, we further design olfactory conditioning experiments with *Drosophila* to assess whether biological generalization similarly benefits from this balance. We find that either increasing the spacing between repetitions or introducing variability in training cues significantly improves test performance in scenarios that differ slightly from training, suggesting that biological generalization also benefits from the structured integration of variations over a certain period of time. These findings demonstrate a shared computational principle underlying biological and artificial systems: structured exposure to appropriate variations promotes generalization from previous experiences. This principle opens up promising directions for designing biologically grounded training paradigms in ANNs while offering a computational lens for understanding naturally evolved "algorithms" in biological brains.

RESULTS

Spaced temporal ensemble of encoding variability in biological and artificial systems

The encoding variability theory in BI posits that generalization is enhanced by appropriately spaced repetitions that introduce diverse representations, through either external inputs or internal neural states.[7,26,27] A parallel mechanism exists in AI where EL improves generalization by aggregating appropriately diversified model variants that capture complementary aspects of the data distribution. Such diversity can arise from input variations introduced through external data augmentation[41] or from innate variations introduced throughout training. The innate variations are typically instantiated via temporal ensemble strategies such as dropout,[35] WA,[36] and KD,[38] which operate at the neuronal, synaptic, and network levels, respectively.

In both biological and artificial systems, the effectiveness of variability in improving generalization critically depends on its magnitude and the temporal spacing over which it is integrated. This observation leads us to hypothesize a shared computational principle: structured exposure to appropriate variations over a certain period of time enhances generalization from training to test. To evaluate this, we systematically investigate how input and innate variations, modulated by controllable spaced intervals, affect generalization performance in ANNs and further examine whether analogous effects occur in

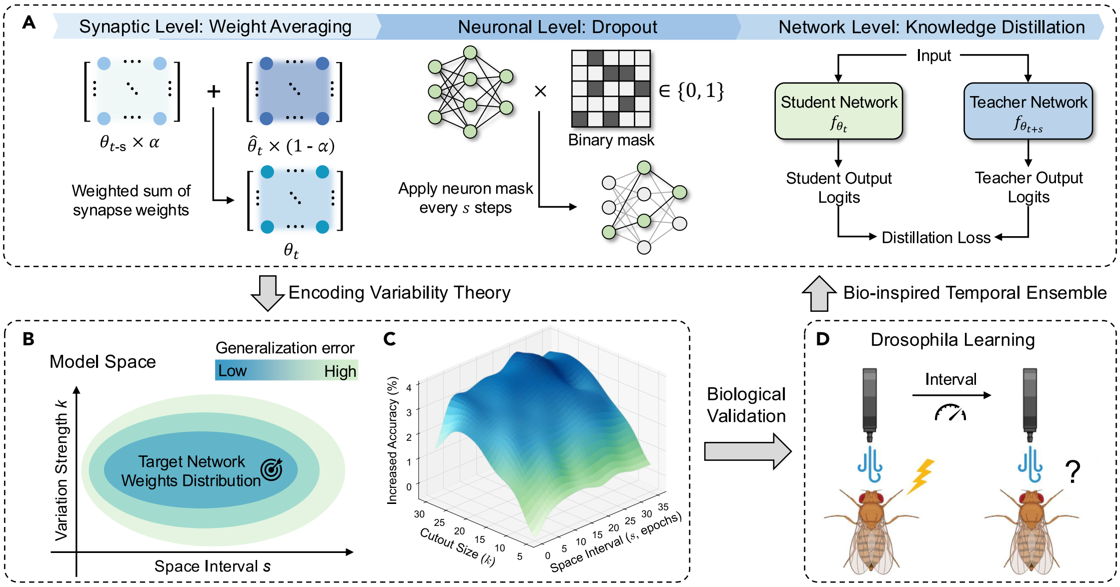

Figure 1. Overview of the spacing effect in biological and artificial systems

(A) Schematic illustration of temporal ensemble strategies at the neuronal, synaptic, and network levels in ANNs, capturing the temporally evolving nature of biological learning.

(B) Two key factors, variation strength and spaced interval, jointly modulate generalization performance in an independent and complementary manner.

(C) Joint analysis of variation strength and spaced interval with cutout-based augmentation on CIFAR-10 dataset reveals an inverted U-shaped performance profile with respect to both factors. All results are averaged over three runs with different random seeds.

(D) *Drosophila* learning with olfactory conditioning experiments.

biological learning and memory through behavioral experiments in *Drosophila*.

Spacing effect with input and innate variations enhances generalization in ANNs

We begin by investigating the spacing effect in ANNs using externally imposed input variations. Specifically, we adopt the cutout augmentation strategy,[42] which simulates environmental perturbations by randomly masking square patches in input images. This strategy allows independent manipulation of two key factors: the variation strength, defined by the cutout size (Figure S1A), and the spaced interval, defined by the frequency at which the cutout size is changed during training (Figure S1B). In ANNs, we use "spaced interval" to denote an interval in training steps or epochs between variability updates (e.g., changing cutout size) rather than a physical delay between learning events as in behavioral experiments. For all ANN experiments, we follow the standard image classification protocol: models are trained on the official training set and evaluated on a disjoint held-out test set (no overlapping images) drawn from the same underlying distribution. Accordingly, "generalization" here refers to training-to-test generalization within each benchmark dataset. To further contextualize this setting, we quantify similarity between the training and test sets using the Wasserstein distance metric (Figure S2). See discussion for an extensive comparison of the generalization concept between biological learning and machine learning.

Using a 4-layer convolutional neural network (CNN) trained on the CIFAR-10 dataset,[43] we investigate how test performance varies with cutout size under a fixed augmentation schedule. We observe a clear inverted U-shaped trend: moderate masking enhances generalization from training to test, whereas overly large occlusions impair the performance (Figure S1C). Next, we introduce temporal structure by periodically altering the cutout size across training epochs. Such a spaced version of cutout augmentation further improves test performance and again yields an inverted U-shaped trend, now with respect to the spaced interval (Figure S1D). Notably, the effects of variation strength and spaced interval appear largely independent and additive, suggesting that both factors contribute complementary benefits to generalization. When combined, they jointly promote more robust representations and improved test performance (Figure 1C).

Recent theoretical work[44] has demonstrated that representative temporal ensemble strategies,[33,34] including dropout,[35] EMA,[37] online KD,[38] and self-KD,[40] are fundamentally equivalent in improving generalization, and all amount to integrating properly diversified model variants throughout training (Figure 1B). Building upon this foundation, we introduce a unified formulation that explicitly organizes temporal ensemble strategies under the common principle of spaced ensembling, governed by the spaced interval s between ensemble updates and the variation strength k applied at each update:

$$\mathcal{H}_t = \mathcal{F}(k \odot \mathcal{H}_{t-s}). \qquad \text{(Equation 1)}$$

$\mathcal{H}_t$ denotes the internal state at the current training step t, which may refer to neuron activations h_t, network parameters θ_t, or model outputs $f_{\theta_t}(x)$ given inputs x. The operator $\odot$ represents element-wise modulation. $\mathcal{F}$ is an ensembling function adapted to each strategy. Throughout the paper, t indexes the training

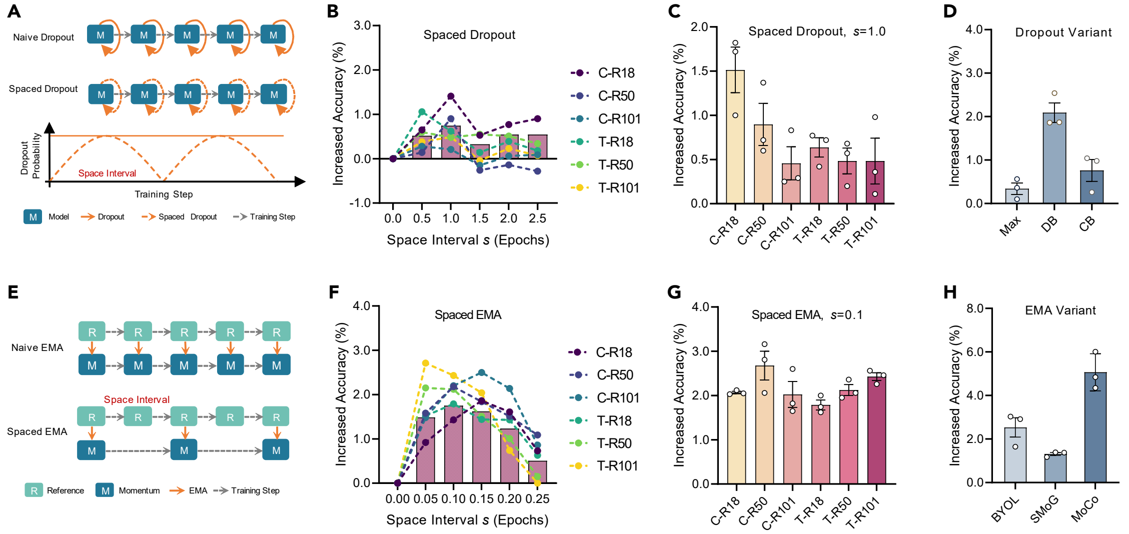

Figure 2. Spacing effect enhances generalization through structured dropout and WA

(A) Spaced version of dropout, where the dropout probability varies periodically to introduce structured neuronal variability during training.

(B) Performance gains under different structured dropout probabilities exhibit an inverted U-shaped trend, indicating optimal performance at intermediate variation strengths. The spaced interval s = 0 denotes the baseline performance without spacing effect modification.

(C) Performance gains of spaced dropout across different network architectures and benchmark datasets.

(D) Spaced version of dropout improves performance in advanced dropout variants, including MaxDropout,[45] DropBlock,[46] and Checkerboard.[47]

(E) Spaced version of EMA, where momentum updates occur at fixed intervals to control synaptic variability.

(F) Performance gains under different momentum spacing intervals also exhibit an inverted U-shaped trend, highlighting the importance of optimal temporal spacing. The spaced interval s = 0 denotes the baseline performance without spacing effect modification.

(G) Performance gains of spaced EMA across different network architectures and benchmark datasets.

(H) Spaced version of EMA improves performance in advanced EMA variants with self-supervised learning, including BYOL,[48] SMoG,[49] and MoCo.[50]

C, CIFAR-100; T, Tiny-ImageNet; R18, ResNet-18; R50, ResNet-50; R101, ResNet-101. All results are averaged over three runs with different random seeds. Data are presented as the mean ± SEM. See Table S1 for the original results of baselines and spaced variants.

steps or epochs and s denotes the interval along the training trajectory between ensemble updates rather than a physical inter-stimulus time. Here, k serves as a possibly high-dimensional variation operator that may encode both the magnitude and structure of variations (e.g., cutout masks, dropout masks, or teacher-student pairing). In our controlled sweeps, we summarize this operator by a scalar "variation strength" for clarity. With this unified formulation, we empirically investigate the bio-inspired spacing effect in ANNs across three levels of innate variations.

Neuronal level: Dropout

Dropout[35] introduces innate variations by randomly deactivating a subset of neurons throughout training. Here, the variation strength corresponds to the dropout rate (i.e., the probability of deactivation), whereas the spaced interval is inherently fixed, as dropout is often applied at every training step. To explore the impact of temporal structure, we employ a periodic schedule for the dropout rate as a spaced version of dropout (Figure 2A; Note S1), akin to the idea of implementing the spacing effect with cutout augmentation. Here, the spaced interval s is implemented as the period in training steps or epochs over which the dropout probability is modulated. The training remains continuous, without inserting physical gaps between mini-batches.

We observe a clear inverted U-shaped relationship between test performance and dropout rate (Figure 2B): mild deactivation promotes diverse activations and therefore improves generalization, whereas excessive sparsity of neuronal activations impairs the model's learning capacity. Importantly, this inverted U-shaped trend persists when jointly manipulating the spaced interval s and the dropout rate as the variation strength k (Figure S3A), mirroring the spacing effect observed in input variations. The spaced version of dropout consistently outperforms standard dropout (Figure 2C) across multiple benchmark datasets (CIFAR-100 and Tiny-ImageNet) and network architectures (ResNet-18, -50, and -101). The benefits generalize across advanced dropout variants, including DropBlock,[46] MaxDropout,[45] and Checkerboard[47] (Figure 2D). These results underscore the robustness of temporally structured neuronal variations in improving generalization.

Synaptic level: WA

WA,[36] such as the EMA,[37] enhances generalization by aggregating temporally spaced parameter snapshots throughout training. Here, the spaced interval is implicitly determined by the EMA momentum coefficient, which controls the effective temporal distance between aggregated snapshots (Figure 2E; Note S2). In this context, the temporal distance refers to the separation between aggregated parameter states along the training steps or epochs rather than elapsed physical time between learning events. The variation strength corresponds to the magnitude of parameter updates, which is not directly tunable in standard EMA.

CellPress
OPEN ACCESS

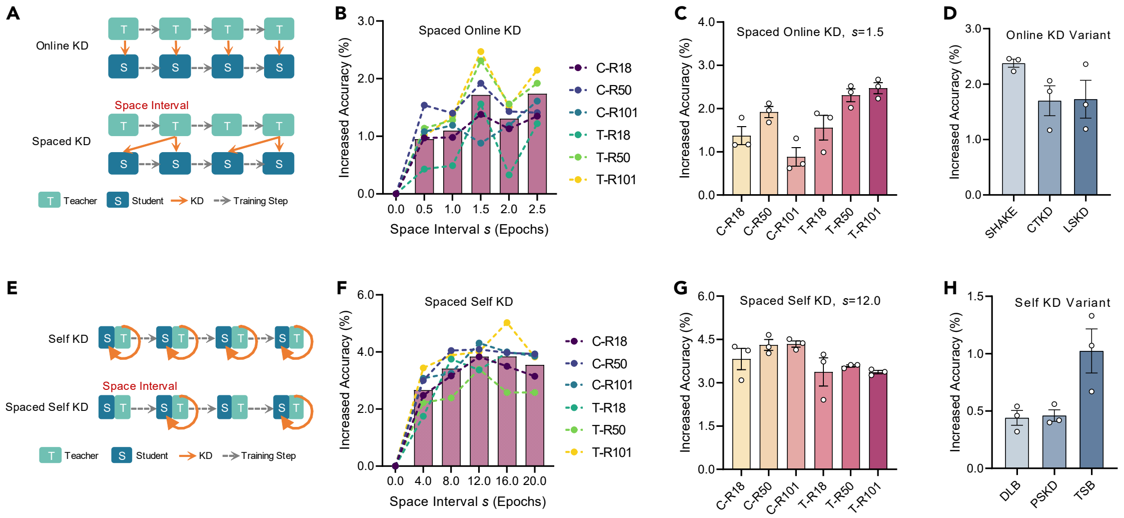

Figure 3. Spacing effect enhances generalization in online KD and self-KD

(A) Spaced version of online KD, where the teacher model is updated at fixed intervals to induce innate variations.

(B) Performance gains of spaced online KD exhibit an inverted U-shaped trend with respect to the spaced interval. The spaced interval $s = 0$ denotes the baseline performance without spacing effect modification.

(C) Performance gains of spaced online KD across different network architectures and benchmark datasets.

(D) Spaced version of online KD improves performance in advanced online KD variants, including SHAKE,[51] CTKD,[52] and LSKD.[53]

(E) Spaced version of self-KD, where the deepest layers (teacher) periodically supervise shallower layers (students) with fixed intervals.

(F) Performance gains of spaced self-KD exhibit an inverted U-shaped trend with respect to the spaced interval. The spaced interval $s = 0$ denotes the baseline performance without spacing effect modification.

(G) Performance gains of spaced self-KD across different network architectures and benchmark datasets.

(H) Spaced version of self-KD improves performance in advanced self-KD variants, including DLB,[54] PSKD,[55] and TSB.[56]

C, CIFAR-100; T, Tiny-ImageNet; R18, ResNet-18; R50, ResNet-50; and R101, ResNet-101. All results are averaged over three runs with different random seeds. Data are presented as the mean ± SEM. See Table S1 for the original results of baselines and spaced variants.

By modulating the momentum value, we again observe a clear inverted U-shaped relationship between test performance and spaced interval (Figure 2F): overly short intervals produce highly similar snapshots with limited diversity, while overly long intervals lead to incoherent aggregation that compromises performance. As shown in Figure S3B, different momentum values (here serving as the variation strength k) substantially affect the location of the performance peak: lower momentum accelerates diversity but impairs stability, whereas higher momentum suppresses useful variations. The spaced version of EMA achieves consistent performance gains across multiple benchmark datasets and network architectures (Figure 2G) and extends to more advanced EMA variants such as BYOL,[48] SMoG,[49] and MoCo[50] (Figure 2H). These results validate the benefits of temporally spaced integration of encoding variability at the synaptic level.

Network level: KD

KD[38] improves generalization by training student models with the soft outputs of more advanced teacher models. We investigate two representative temporal variants: online KD,[39] where the teacher is asynchronously updated throughout training, and self-KD,[40] where the student periodically supervises itself using earlier snapshots. In both cases, the spaced interval corresponds to the number of epochs between teacher updates, while the variation strength naturally arises from the divergence between teacher and student over time. Therefore, the spaced interval in KD is implemented as an update frequency for teacher-student alignment rather than elapsed physical time between learning events.

For online KD (Figure 3A; Note S3), we observe a consistent inverted U-shaped relationship between test performance and spaced interval (Figure 3B): overly short intervals offer limited variation, while overly long intervals lead to teacher-student mismatch and degraded performance. A similar trend emerges in self-KD (Figures 3E–3H; Note S4), where periodic re-alignment with earlier snapshots again exhibits an inverted U-shaped performance profile (Figure 3F). Further, Figure S3C confirms that adapting the variation strength (e.g., via data shuffling) affects the optimal spaced interval, with stronger variations favoring shorter intervals. The spaced versions of online KD (Figures 3C and 3D) and self-KD (Figures 3G and 3H) yield consistent performance gains across multiple benchmark datasets, network architectures, and advanced variants (e.g., SHAKE,[51] CTKD,[52] and LSKD[53] for online KD and DLB,[54] PSKD,[55] and TSB[56] for self-KD), suggesting the effectiveness of bio-inspired spacing effect at the network level.

Together, these results demonstrate that appropriately spaced exposure to either input or innate variations, whether introduced through data augmentation,[42] dropout,[35] WA,[36] online KD,[38] or self-KD,[40] consistently enhances generalization from training to test in ANNs. Moreover, Figure S3 highlights a consistent inverted U-shaped relationship between variation strength k and spaced interval s across all methods, reinforcing the unified role of spacing-induced encoding variability from a machine learning perspective.

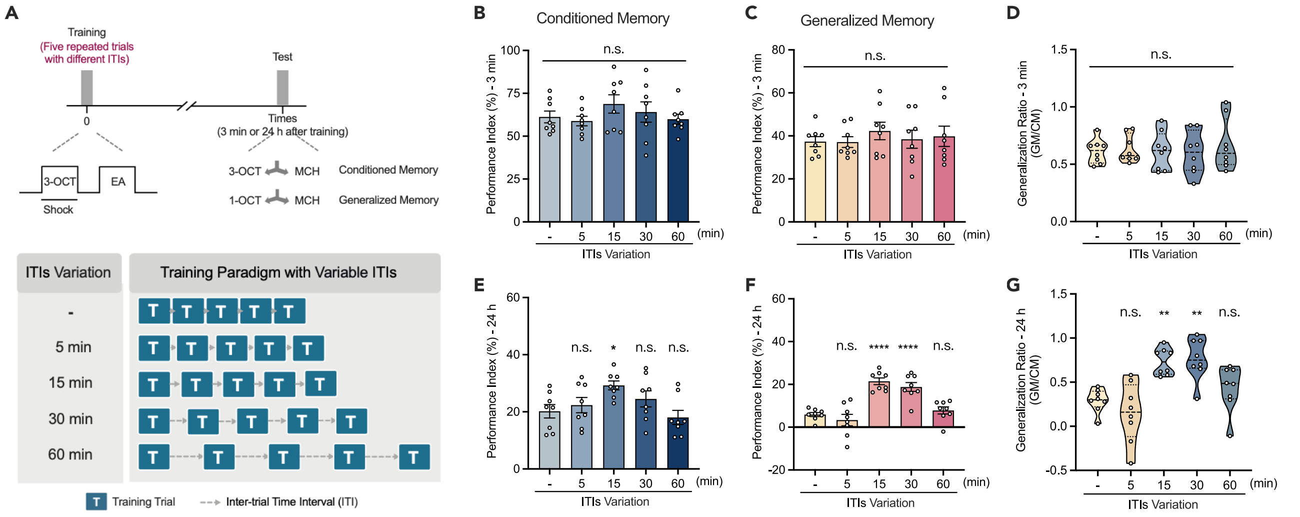

Figure 4. Spaced training enhances memory generalization in *Drosophila*

(A) Experimental paradigm for aversive olfactory conditioning with variable inter-trial intervals (ITIs). The massed training group (−) receives five trials with a 45-s ITI, while the spaced training groups (5, 15, 30, and 60 min) receive trials with increasing ITIs. Conditioned and generalized memory are tested by presenting the CS+ odor (3-OCT) or a structurally similar odor (1-OCT), respectively, against a novel odor (MCH).

(B and C) Conditioned memory (B, $n = 8$) and generalized memory (C, $n = 8$) at 3 min show no significant difference across ITI groups.

(D) The generalization ratio shows no significant difference across ITI groups at 3 min ($n = 8$).

(E and F) Conditioned memory (E, $n = 8$) and generalized memory (F, $n = 8$) at 24 h exhibit an inverted U-shaped pattern across ITI groups.

(G) The generalization ratio is significantly improved in the 15- and 30-min ITI groups at 24 h ($n = 8$).

Data are presented as the mean ± SEM. Statistical significance is determined by one-way ANOVA with Dunnett's multiple comparisons test. $^*p \leq 0.05$, $^{**}p \leq 0.01$, $^{****}p \leq 0.0001$, and n.s., not significant.

Additionally, we examine whether spacing effects at different levels are complementary by evaluating combinations of the spaced variants of dropout, EMA, and KD. We observe cumulative performance gains when combining spaced dropout and spaced EMA relative to using either strategy alone. This suggests that imposing spacing concurrently at the neuronal and synaptic levels introduces partially non-overlapping sources of variation that further improve generalization. However, we note that the spaced KD condition does not yield further additive benefits when combined with these other spacing effects (Figure S4). To test whether the performance gains arise from the periodic structure of spacing, we compare the periodic spaced schedules against matched random schedules in which the same variability updates are applied at irregular, shuffled intervals while preserving the same average update frequency (Figure S5). The periodic spaced schedules consistently outperform the random counterparts across temporal ensemble strategies, benchmark datasets, and network architectures. These results suggest the periodic structure of spacing plays an important role in improving generalization, consistent with the precise timing requirements observed in biological spacing effects.[5,7,20]

Finally, we characterize how spacing influences the training trajectory. Across temporal ensemble strategies, spaced training does not noticeably increase early-stage learning speed, but it consistently raises the converged performance ceiling (Figure S6), leading to higher final test accuracy. These improvements remain consistent under different magnitudes of parameter perturbations by adding Gaussian noise (Figure S7), suggesting the robustness of our parameterized solution in adapting to changes.

Spaced training and trial-to-trial variation enhance generalization in *Drosophila*

To investigate whether the unified role of spacing-induced encoding variability in ANNs also applies to biological systems, we train *Drosophila* fruit flies using a classical olfactory aversive conditioning paradigm and assess both conditioned memory and its generalization performance[57–59] (Figures S8A and S8B). In a forward training (FT) trial, flies are exposed to the odor 3-octanol (3-OCT) paired with electric shocks (conditioned stimulus plus [CS+]), followed by the odor ethyl acetate (EA) without shock (conditioned stimulus minus [CS−]). Both the odor exposure only (OEO) trial and the backward training (BT) trial serve as controls for evaluating associative memory performance. After training, conditioned and generalized memory are tested by presenting either the CS+ odor (3-OCT) or a structurally similar odor (1-octanol [1-OCT]), respectively, against a novel odor (4-methylcyclohexanol [MCH]). Our experiments validate that memory formation is associative and that flies can generalize to a similar odor as early as 3 min after training (Figure S8C–S8E).

First, we examine the effect of temporal spacing on memory generalization. We train flies with five repeated trials separated by different inter-trial intervals (ITIs), ranging from massed training (45-s ITI) to spaced training (5-, 15-, 30-, or 60-min ITI; Figure 4A). At 3 min after training, all groups exhibit robust conditioned and generalized memory, with no significant differences across variable ITI conditions (Figures 4B and 4C). The generalization ratio also has no significant difference among groups (Figure 4D). In contrast, at 24 h after training, both conditioned and generalized memory are significantly influenced by ITI variation. Specifically, flies subjected to spaced training with 15-min

ITIs show significantly enhanced conditioned and generalized memory compared to the massed training group (Figures 4E and 4F), resulting in an inverted U-shaped pattern across ITI groups (Figure 4G). These results underscore the importance of spaced intervals in promoting memory generalization.

Next, we investigate the impact of encoding variability, inspired by our findings in machine learning. We introduce trial-to-trial variation during massed training by systematically varying the odor delivery flow rate (0.5, 1, or 1.5 K mL/min) across five trials (Figure 5A). This manipulation does not impair conditioned memory, as all variable-training groups performed comparably to the constant-flow control group (Figure 5B). Strikingly, introducing variability significantly enhances generalized memory (Figure 5C) and increases the generalization ratio (Figure 5D). This benefit is also persistent, as the enhancement in generalized memory is still evident 24 h after training (Figures 5E and 5F). However, varying shock intensities across trials does not affect generalization under these conditions (Figure S9), indicating that variability in the conditioned sensory input (CS), rather than variability in the reinforcement strength (US), is the critical factor driving improved generalization.

To further explore the interplay between encoding variability and classical biological spaced learning, we examine whether trial-to-trial variation interacts with ITIs (Figure 5G). Beyond the benefits of encoding variability observed during massed training (45-s ITIs), we find that extending the ITIs to 15 min also significantly enhances memory generalization. Remarkably, combining 15-min ITIs and trial-to-trial variation yields a synergistic improvement in both generalized memory and the generalization ratio (Figures 5I and 5J), surpassing the effects of either manipulation alone. This additive enhancement occurs without compromising the primary conditioned memory (Figure 5H), suggesting that encoding variability and temporal spacing may engage complementary pathways underlying memory generalization. Using the protein synthesis inhibitor cycloheximide (CXM), we find that while the 24-h conditioned memory after spaced training is strongly reduced, confirming its reliance on classical long-term memory pathways,[7,20] the generalization enhancement triggered by encoding variability remains largely preserved (Figure S10).

Together, the biological findings in *Drosophila* are consistent with the computational results in ANNs. Both appropriately spaced training intervals and increased trial-to-trial sensory variability can significantly and lastingly enhance the generalization of memories. These results demonstrate a convergent computational principle of biological and artificial systems in which structured exposure to temporal or contextual variations promotes the formation of more robust and generalizable representations.

DISCUSSION

In this study, we identify an encoding-variability-inspired principle[26–28] as a shared computational principle that enhances generalization in both biological and artificial systems. Specifically, we demonstrate that appropriately spaced exposure to input or innate variations significantly improves generalization from training to test in ANNs (Figures 2 and 3). This effect is robust across a variety of benchmark datasets, network architectures, and temporal ensemble strategies. Importantly, we find that the two key factors, variation strength and spaced interval, independently and additively enhance generalization. Beyond artificial systems, behavioral experiments in *Drosophila* reveal similar benefits under spaced exposure to variable training cues, showing that both spaced training intervals (Figure 4) and increased sensory variations (Figure 5) enhance memory generalization in olfactory associative learning. Together, these findings demonstrate how computational principles derived from ANNs can guide biological experimentation, suggesting that encoding variability may operate as a unified mechanism that enhances generalization across both artificial and natural learners.

A central concept in our work is generalization. In machine learning, this typically refers to a model's ability to apply knowledge from training data to unseen test data drawn from the same underlying distribution.[1] In neuroscience, although animals are often tested with the same sensory cues presented during training, it is rarely possible to exactly reproduce the internal states or environmental conditions across training and testing episodes. As a result, what is traditionally studied as precise memory in neuroscience implicitly involves a form of generalization.[4,60]

While the spacing effect has classically been linked to protein-synthesis-dependent long-term memory, our results suggest that memory persistence and memory flexibility can show different sensitivities to protein synthesis inhibition. Under CXM treatment, conditioned memory after spaced training is strongly reduced, whereas the generalization enhancement induced by sensory variability remains largely preserved (Figure S10). This pattern differs from previously reported "rapid generalization" mechanisms,[61] which rely on prior associative linking between environments. Instead, our results align with a consolidation of variable experiences that, under our experimental conditions, appear less sensitive to protein synthesis inhibition. Nonetheless, we acknowledge that a more potent or prolonged inhibition might potentially impair generalized memory as well.[62] This suggests that while temporal spacing is essential for memory stabilization, the "flexibility" of memory can be modulated by sensory variability, potentially through distinct molecular or circuit-level changes. Therefore, biological learning may utilize parallel mechanisms to balance memory specificity and generalization, a principle that aligns with the dual requirements of adaptive learning systems in both biological and computational contexts.

Notably, the enhancement of generalized memory emerges after 24 h but not at 3 min post-training (Figure 4), suggesting that spaced learning facilitates the delayed consolidation and gradual refinement of acquired knowledge into new, related contexts, without requiring pre-configured associations. We suggest that this type of exact recall in biological learning (despite identical cues) is more analogous to the concept of generalization in machine learning (i.e., from training data to similar test data), while the broader notion of generalization in biological learning (e.g., transferring across different cues or contexts) parallels the more difficult domain generalization tasks in machine learning (i.e., from training data to dissimilar test data). Our work bridges this conceptual gap by showing how spacing and variability jointly support generalization in both fields.

The spacing effect has long been recognized in neuroscience for its beneficial impact on memory formation, but its

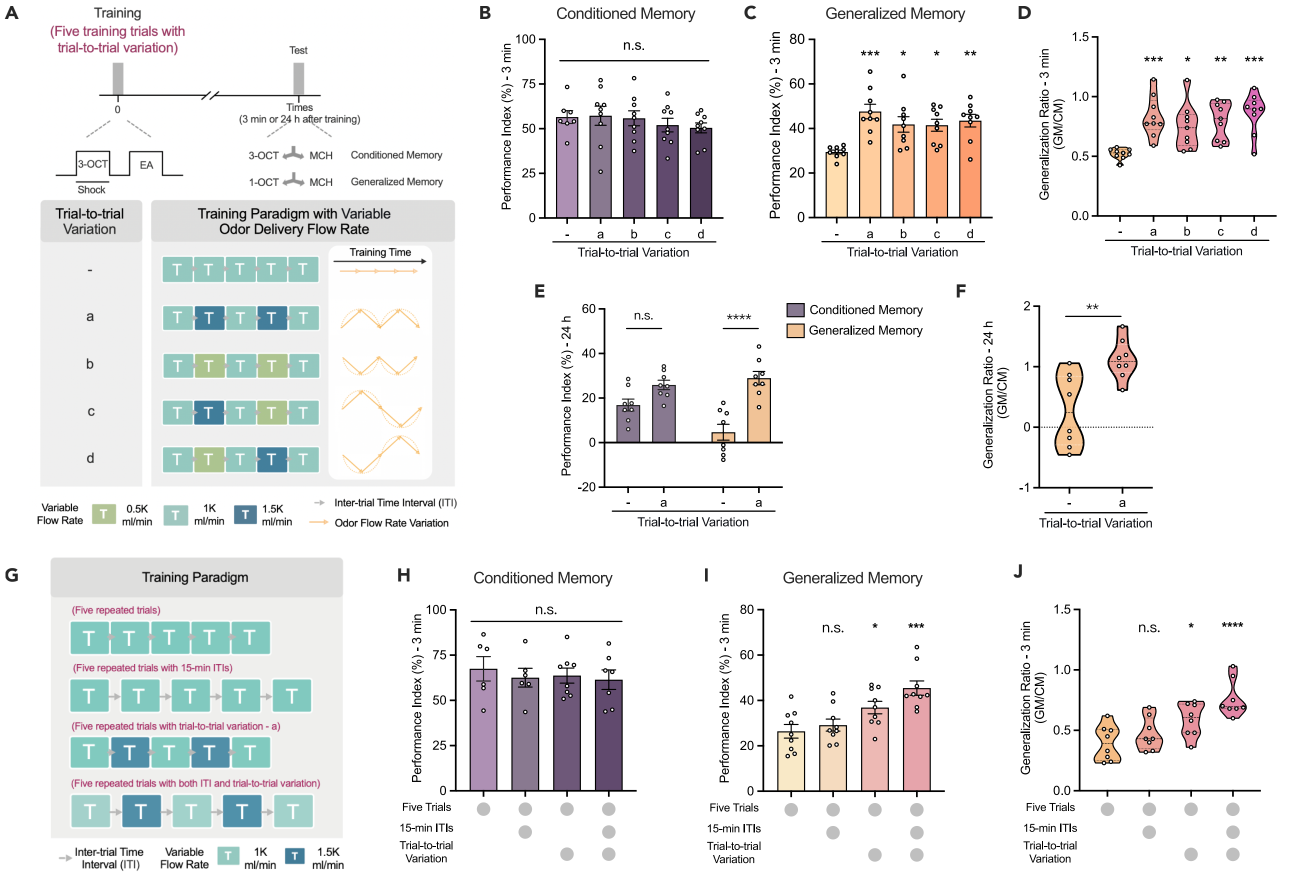

Figure 5. Trial-to-trial variation enhances memory generalization in *Drosophila*

(A) Experimental paradigm for massed training with variable sensory input. The control group (–) receives five trials with a constant odor delivery flow rate, while the experimental groups (a–d) receive trials with varying flow rates to introduce encoding variability.
(B) Conditioned memory shows no significant difference between control and experimental groups (a–d) at 3 min (n = 7–9).
(C) All experimental groups (a–d) exhibit enhanced generalized memory at 3 min (n = 9).
(D) The generalization ratio is significantly increased in experimental groups (a–d) at 3 min (n = 9).
(E) Generalized memory remains enhanced in the experimental group (a) compared to the control group (–) at 24 h (n = 8).
(F) The generalization ratio remains elevated in the experimental group (a) at 24 h (n = 8).
(G) Experimental paradigm for evaluating the interaction between temporal spacing and trial-to-trial variation: five repeated trials (massed), five trials with trial-to-trial variation, five trials with 15-min ITIs (spaced), and five trials combining both 15-min ITIs and trial-to-trial variation.
(H) Conditioned memory shows no significant difference across all groups at 3 min (n = 5).
(I and J) Generalized memory (I) and generalization ratio (J) at 3 min.
Data are presented as the mean ± SEM. Statistical significance is determined by one-way ANOVA with Dunnett's multiple comparisons test. *$p \leq 0.05$, **$p \leq 0.01$, ***$p \leq 0.001$, ****$p \leq 0.0001$, and n.s., not significant.

computational implications for AI remain underexplored. Our results demonstrate that incorporating spacing mechanisms, particularly through temporal ensemble strategies that mimic the temporally evolving nature of biological learning, can substantially enhance test performance in ANNs. Unlike traditional EL methods that require training multiple models in parallel, temporal ensemble strategies efficiently aggregate internal variations over time. By further introducing structured spacing into these methods, we unlock new performance gains while maintaining computational efficiency. These insights have practical implications for a range of AI challenges, including few-shot learning,[63–65] continual learning,[66–69] and adversarial robustness,[70,71] where generalization from limited or noisy data is critical.

From the perspective of biological learning, prior studies have often manipulated either variation strength or spaced interval in isolation. For instance, encoding variability is typically studied by altering environmental conditions while keeping temporal spacing fixed, whereas spacing effects are evaluated by varying ITIs with constant stimuli.[26–28] In our ANN experiments, training proceeds continuously, and the spaced interval is implemented computationally as an interval along the training steps or epochs between ensemble updates (e.g., changing cutout size, modulating dropout probability, aggregating network states, or updating teachers in KD). This design allows joint manipulation of variation strength and spaced interval to study their combined effects on generalization. Inspired by temporal ensemble strategies,[30,31,33,34] this dual modulation provides a more

comprehensive understanding of the importance of encoding variability. The observed complementary effect between variation strength and spaced interval highlights their distinct, additive benefits to generalization, which may inform future designs of biological learning and memory experiments.

A limitation of our current formulation is that we primarily characterize variations using a scalar variation strength k with a spaced interval s. In practice, variations are often multi-dimensional, and their detailed structure (e.g., specific masking patterns or teacher-student pairings) may further influence diversity and generalization. A systematic analysis of how structured variations interact with the spaced interval is an important direction for future work. While our computational experiments demonstrate robust performance gains, the current link between the spacing effect and generalization in ANNs is supported primarily by performance-level evidence. Future work is expected to conduct more direct analyses that quantify representational diversity and internal network dynamics for a mechanistic interpretation. In addition, while we emphasize an encoding-variability-inspired perspective for its connection to generalization and temporal ensembling, deficient-processing and study-phase retrieval mechanisms may also contribute to the spacing effect depending on task and memory phase. Furthermore, while our biological experiments demonstrate the behavioral benefits of training variability, we have not yet directly characterized the underlying coding variability at the neuronal level. Our current findings provide a bio-inspired, computational-to-behavioral validation of the encoding variability theory, but the specific neural dynamics, such as how synaptic ensembles or population activities evolve across spaced intervals, require further investigation across multiple biological levels.

Looking ahead, this study exemplifies the promise of NeuroAI,[1,3,4] an emerging field that seeks to accelerate progress in both neuroscience and AI through mutual inspiration. Our findings demonstrate how biologically grounded learning principles, such as spacing and encoding variability, can be translated into algorithmic gains for ANNs, while controlled experiments in ANNs can, in turn, generate testable hypotheses about biological learning mechanisms. Promising future directions include developing adaptive spacing schedules that dynamically adjust based on learning progress, extending multi-modal generalization across visual, auditory, and other sensory modalities, and integrating bio-inspired plasticity rules to further align ANNs with the dynamics of biological learning and memory. These directions may pave the way toward a unified and mechanistic understanding of generalization across both biological and artificial systems.

METHODS

Spacing effect of temporal ensemble

We investigate how the spacing effect in biological learning can be computationally leveraged to enhance generalization in ANNs via temporal ensemble strategies. Specifically, we instantiate this principle in three representative methods, WA, dropout, and KD, which operate at the synaptic, neuronal, and network levels, respectively. Our central hypothesis is that the generalization benefits observed in biological systems through spaced repetition and encoding variability have computational analogs in ANNs, wherein variation strength and spaced intervals jointly shape learning outcomes.

Problem formulation

Let $\mathcal{D}_{\text{train}} = \{(x_i, y_i)\}_{i=1}^{N_{\text{train}}}$ and $\mathcal{D}_{\text{test}} = \{(x_i, y_i)\}_{i=1}^{N_{\text{test}}}$ denote the training and test sets drawn from the same underlying distribution, where each sample consists of an input-label pair. A neural network model $f_\theta(x)$ parameterized by θ is trained to minimize a supervised loss, defaulting to the cross-entropy (CE) loss for classification tasks:

$$\mathcal{L}(\theta; x, y) = -\sum_{c=1}^{C} y_c \log(f_\theta(x)_c), \qquad \text{(Equation 2)}$$

where $f_\theta(x)_c$ is the predicted probability for class c and y_c is a one-hot-encoded ground-truth label.

The model's generalization performance $\mathcal{G}$ is evaluated on $\mathcal{D}_{\text{test}}$ using the average top-1 accuracy:

$$\mathcal{G}(\theta; \mathcal{D}_{\text{test}}) = \frac{1}{N_{\text{test}}} \sum_{i=1}^{N_{\text{test}}} \mathbb{I}\left[\operatorname*{argmax}_c f_\theta(x_i)_c = y_i\right], \qquad \text{(Equation 3)}$$

where $\mathbb{I}[\cdot]$ is the indicator function, returning 1 if the predicted class matches the true label and 0 otherwise. $f_\theta(x_i)_c$ denotes the predicted probability for class c on test input x_i.

Unified temporal ensemble framework

Building upon prior theoretical efforts on temporal ensemble strategies,[33,34,44] we introduce a unified formulation that captures the core components of the spacing effect with two variables: spaced interval s and variation strength k. The ensemble update process can be summarized as

$$\mathcal{H}_t = \mathcal{F}(k \odot \mathcal{H}_{t-s}), \qquad \text{(Equation 4)}$$

where $\mathcal{H}_t$ denotes the internal state at the current training step t, which may refer to neuron activations h_t, network parameters θ_t, or model outputs $f_{\theta_t}(x)$ given input x. The operator $\odot$ represents element-wise modulation. $\mathcal{F}$ is an ensembling function adapted to each strategy. In general, k may represent a structured operator (e.g., a mask or transformation) rather than a single scalar. Throughout the paper, we use variation strength as a scalar summary to enable systematic comparisons, while the detailed structure of variations may further modulate diversity and performance. The following sections define these instances and describe how the bio-inspired spacing effect is implemented in each case.

Dropout

Dropout[35] operates at the neuronal level by introducing stochastic perturbations to the neuronal activations. Let $\hat{h}_t$ denote the pre-activation vector at training step t. In the standard dropout formulation, a binary dropout mask $r_t \sim \text{Bernoulli}(p_t)$ is sampled element-wise with dropout rate $p_t \in [0,1]$ and applied as

$$h_t = r_t \odot \hat{h}_t, \qquad \text{(Equation 5)}$$

where h_t denotes the post-dropout neuronal activations and $\odot$ denotes element-wise multiplication.

In our unified framework, the internal state at training step t is defined as $\mathcal{H}_t = h_t$, with temporal ensembling introduced throughout training:

$$\mathcal{H}_t = \mathcal{F}(k \odot h_{t-s}), \quad \text{(Equation 6)}$$

where s is the spaced interval, k corresponds to the dropout mask $r_{t\text{-}s}$ from a previous training step, and $\mathcal{F}(\cdot)$ is a strategy-specific ensembling function (e.g., averaging or consistency regularization).

To further exploit the bio-inspired spacing effect, we modulate the dropout rate p_t periodically throughout training (Note S1). Specifically, we define

$$p_t = p_{\max} \cdot \sin\left(\frac{2\pi t}{s}\right), \quad \text{(Equation 7)}$$

where $p_{max} \in [0,1]$ is the maximum dropout rate and s controls the period of modulation. This introduces structured variability in the dropout-induced representations, enabling both temporal spacing and controlled variation strength to improve generalization.

WA

WA[36] operates at the synaptic level by aggregating parameter snapshots throughout training. Let $\widehat{\theta}_t$ denote the original parameters updated by gradient descent at training step t and θ_t represent the ensembled parameters. In the standard EMA formulation, the ensemble is updated iteratively as

$$\theta_t = \alpha\theta_{t-1} + (1-\alpha)\widehat{\theta}_t, \quad \text{(Equation 8)}$$

where $\alpha \in [0,1]$ is the momentum coefficient controlling the influence of past parameters. The underlying base parameters evolve via standard gradient descent

$$\widehat{\theta}_t = \widehat{\theta}_{t-1} - \eta\nabla\mathcal{L}_{\text{task}}(f_{\widehat{\theta}_t}(x), y), \quad \text{(Equation 9)}$$

with learning rate η and task loss $\mathcal{L}_{\text{task}}$.

In our unified framework, the internal state is defined as $\mathcal{H}_t = \theta_t$, and the ensembling process with spacing becomes

$$\mathcal{H}_t = \mathcal{F}(k \odot \theta_{t-s}), \quad \text{(Equation 10)}$$

where s is the spacing interval, k corresponds to the momentum coefficient, and $\mathcal{F}(\cdot)$ denotes the EMA update.

To incorporate the spacing effect, we propose a spaced version of EMA (Note S2) in which parameter aggregation occurs every s steps:

$$\theta_t = \begin{cases} \alpha\theta_{t-s} + (1-\alpha)\widehat{\theta}_t, & \text{if } t \bmod s = 0, \\ \theta_{t-s}, & \text{otherwise.} \end{cases} \quad \text{(Equation 11)}$$

This modification introduces explicit temporal spacing into the ensemble dynamics, allowing the model to integrate more diverse parameter states and improving robustness via spaced accumulation.

KD

KD[38] operates at the network level by guiding a student model with soft outputs of more advanced teacher models. Among commonly used KD methods, online KD and self-KD naturally align with the concept of temporal ensembling and are suitable for investigating the bio-inspired spacing effect.

Let $f_{\theta_t}(x)$ denote the teacher's output at training step t for input x and $f_{\theta_{t-s}}(x)$ denote the student's output from an earlier checkpoint. In standard online KD, the teacher is a more advanced model (e.g., a later snapshot or moving average), and the student attempts to match the teacher's softened outputs. The distillation loss is defined as

$$\mathcal{L}_{\text{KD}} = \text{KL}(f\theta_{t-s}(x) \parallel f_{\theta_t}(x)), \quad \text{(Equation 12)}$$

where $\text{KL}(\cdot\|\cdot)$ denotes the Kullback-Leibler divergence between teacher and student outputs. The student is trained using a combination of task and distillation losses:

$$\mathcal{L}_{\text{student}} = \mathcal{L}_{\text{task}}(f_{\theta_{t-s}}(x), y) + \lambda\mathcal{L}_{\text{KD}}, \quad \text{(Equation 13)}$$

where λ is a weighting coefficient balancing task supervision and distillation guidance.

In our unified framework, we define the internal state at time step t-s as $\mathcal{H}_{t-s} = f_{\theta_{t-s}}(x)$. The spaced version of online KD introduces temporal ensembling through delayed teacher-student supervision:

$$\mathcal{H}_{t-s} = \mathcal{F}(k \odot f_{\theta_t}(x)), \quad \text{(Equation 14)}$$

where s is the spaced interval, k corresponds to a variation operator modulating the teacher signal (e.g., temperature scaling or input perturbation), and $\mathcal{F}(\cdot)$ is the distillation function (e.g., KL divergence or consistency regularization).

To enforce spacing explicitly, the teacher is updated every s steps based on task supervision:

$$\mathcal{L}_{\text{teacher}} = \mathcal{L}_{\text{task}}(f_{\theta_t}(x), y). \quad \text{(Equation 15)}$$

This temporal offset between teacher and student promotes representational diversity and prevents premature convergence to local minima.

For self-KD,[40] the teacher and student reside within the same network, typically across different layers, heads, or temporal branches. The spaced version of self-KD (Note S4) alternates which internal representations serve as distillation targets over time. The formulation remains consistent within our framework, with spacing applied to internal states rather than model snapshots, allowing earlier components to imitate more refined representations from future computation stages.

Experimental setup

To evaluate the impact of bio-inspired spacing effect on learning and generalization, we design parallel experiments in both biological and artificial systems.

Experiments with ANNs

To simulate input variability analogous to environmental changes in biological experiments, we first employ cutout augmentation[42] on a 4-layer CNN architecture. We then extend our study by incorporating the spacing effect into representative temporal ensemble strategies across synaptic, neuronal, and network levels.

Datasets and training protocols

We evaluate on three benchmark datasets commonly used in image classification tasks: CIFAR-10,[43] CIFAR-100,[43] and Tiny-ImageNet.[72] CIFAR-10 contains 10 classes with 50,000 training

CellPress
OPEN ACCESS

and 10,000 testing images of size 32 × 32. CIFAR-100 contains 100 classes with 50,000 training and 10,000 testing images of size 32 × 32. Tiny-ImageNet contains 200 classes with 100,000 training and 10,000 testing images of size 64 × 64. Experiments are conducted on multiple backbone architectures, including ResNet-18, -50, and -101.[73] To ensure fair comparisons, we adopt identical training protocols following prior work.[40,42,74] Unless otherwise specified, all models are trained for 80 epochs using stochastic gradient descent (SGD) with momentum 0.9, batch size 128, and a constant learning rate of 0.01.

WA

In standard EMA,[37] the momentum model f_{θ_t} is updated at each training step from the reference model $f_{\hat{\theta}_t}$. The spaced version of EMA updates f_{θ_t} only every s steps, explicitly enforcing temporal spacing. The momentum coefficient α is set to 0.99 by default. We further implement the spaced version of EMA within advanced self-supervised learning methods, including BYOL,[48] SMoG,[49] and MoCo,[50] with the official codebase (https://github.com/lightly-ai/lightly). In these methods, $f_{\hat{\theta}_t}$ is updated using contrastive loss, and f_{θ_t} is updated using EMA.

Dropout

The standard dropout[35] introduces stochasticity by randomly zeroing out neuronal activations at each training step. The spaced version of dropout instead modulates the dropout probability periodically using a sinusoidal function parameterized by the spaced interval s and the variation strength k (i.e., the maximum dropout probability p_{max}). We set p_{max} = 0.3 by default. We further implement the spaced version of dropout with advanced dropout variants, including DropBlock,[46] MaxDropout,[45] and Checkerboard Dropout.[47]

KD

In standard online KD,[39,75,76] the teacher model transfers knowledge to the student model at each training step. The spaced version of online KD introduces a spaced interval s (in epochs), such that the teacher is trained for s steps before guiding the student for the next s steps. In the case of self-KD, where deeper layers act as teachers to guide shallower ones at each training step, the spaced version alternates between training the entire network for s steps and then training the shallow layers for s steps using the deepest layers' outputs. Following prior implementation,[40,74] we set the KD temperature to 3.0, the feature loss coefficient to 0.03, and the distillation loss coefficient to 0.3. We further implement the spaced version of more advanced online KD (SHAKE,[51] CTKD,[52] and LSKD[53]) and self-KD (DLB,[54] PSKD,[55] and TSB[56]) variants.

Experiments with biological systems

This section describes the biological experimental setup used to examine the spacing effect in *Drosophila* melanogaster.

Fly strains

All fly strains are reared on standard cornmeal medium under a 12:12 h light/dark cycle at 23°C and 60% humidity. We use wild-type flies of the *W1118* strain, which are aged 2–3 days post-eclosion and include a mixture of males and females for behavioral assays.

Olfactory aversive conditioning

To evaluate associative learning, we perform a classical Pavlovian olfactory conditioning procedure.[57–59] Flies are transferred to a behavioral room for at least 30 min to adapt to the experimental environment. Approximately 80–100 flies are subjected to the following sequential stimuli in a forward conditioning trial: air for 90 s, an odor paired with 12 pulses of a 60 V electric shock (CS+) for 1 min, air for 45 s, a second odor without pairing the electric shock (CS−) for 1 min, and finally air for 45 s. For backward training, the electric shock ends 1 min before the onset of the first CS+ odor. In the OEO control, flies are exposed to both odors in the same sequence but without any electric shock delivery. All behavioral experiments are conducted at 25°C and 60% relative humidity. The following odorants are used, all diluted in mineral oil: MCH (1.0 × 10^{-3} dilution, Fluka), 3-OCT (1.5 × 10^{-3} dilution, Sigma-Aldrich), 1-OCT (2.0 × 10^{-3} dilution, J&K Scientific), and EA (1.0 × 10^{-3} dilution, Alfa Aesar).

Drug feeding

For pharmacological manipulation, flies are fed with 35 mM CXM (Sigma) in a control solution (5% [wt/vol] glucose and 3% [vol/vol] ethanol) to block protein synthesis, while control groups (CXM−) received the vehicle solution only. To inhibit *de novo* protein synthesis, flies were fed 35 mM CXM (Sigma-Aldrich) in vehicle solution (5% [wt/vol] glucose and 3% [vol/vol] ethanol) for 14–16 h prior to massed or spaced training. This pharmacological treatment was maintained throughout the 24-h retention interval until testing. This concentration and feeding protocol has been extensively validated to effectively block protein synthesis in *Drosophila* memory assays.[58,77]

Memory testing and performance index

Memory performance is evaluated using a standard T-maze apparatus 3 min (immediate memory) or 24 h (long-term memory) after training. Flies are given 1 min to choose between the CS+ odor (or a structurally similar analog) and a novel odor. The preliminary performance index (PI) is calculated as

$$PI = \frac{n_{novel} - n_{CS+}}{n_{novel} + n_{CS+}} \times 100, \quad \text{(Equation 16)}$$

where n_{novel} and n_{CS+} denote the number of flies in the novel odor and CS+ arms, respectively. A PI of 100 indicates that all flies make the right choice to avoid the odor paired with the electric shock, while a PI of 0 indicates no memory retention, as reflected by a 50:50 distribution between the arms. To balance naive odor bias, two reciprocal groups are trained and tested simultaneously. One group is trained with CS+/CS− and the other with OEO or backward training (i.e., shock delivered 1 min before CS+ odor exposure). The final learning performance is calculated as a corrected PI, defined by subtracting the PI of the control group from that of the associative training group to account for non-associative components.

RESOURCE AVAILABILITY

Lead contact

Requests for further information and resources should be directed to and will be fulfilled by the lead contact, Liyuan Wang (liyuanwang@tsinghua.edu.cn).

Materials availability

This study did not generate new materials.

Data and code availability

- All benchmark datasets used in this paper are publicly available, including CIFAR-10/100[43] (https://www.cs.toronto.edu/~kriz/cifar.html) and Tiny-ImageNet[72] (https://www.image-net.org/download.php). Statistical analysis is performed using GraphPad Prism software. Data are considered normally distributed if they pass the Shapiro-Wilk test (for $n < 8$) or the Anderson-Darling test (for $n \geq 8$). For normally distributed data, comparisons between the two groups are performed using the two-tailed unpaired t test; comparisons between multiple groups are performed using the one-way ANOVA test followed by Dunnett's multiple comparisons test and the two-way ANOVA test followed by Sidak's multiple comparisons test. Results are reported as n.s. (not significant) $p > 0.05$, $^{*}p < 0.05$, $^{**}p < 0.01$, $^{***}p < 0.001$, and $^{****}p < 0.0001$.
- Our source code is available at GitHub (https://github.com/SunGL001/spacing_generalization) and has been archived at Zenodo.[78]

ACKNOWLEDGMENTS

This work is supported by the STI2030-Major Projects (no. 2022ZD0204900 to Y.Z.), the Beijing Major Science and Technology Project (no. Z251100008425003 to L.W. and Y.Z.), the NSFC Projects (nos. 62406160 to L.W. and 32021002 to Y.Z.), and the National Science and Technology Major Project (no. 2022ZD01163013 to B.L.).

AUTHOR CONTRIBUTIONS

Study conception and design, G.S. and L.W.; computational experiment performing, G.S. and H.Y.; biological experiments, N.H.; visualization and data analysis, G.S., N.H., H.Y., and L.W.; funding acquisition, Y.Z., L.W., and B.L.; results discussion and interpretation, G.S., N.H., H.Y., L.W., J.Z., Q.L., B.L., and Y.Z.; manuscript writing, G.S., N.H., and L.W.; manuscript discussion, G.S., N.H., H.Y., J.Z., Q.L., B.L., Y.Z., and L.W.

DECLARATION OF INTERESTS

The authors declare no competing interests.

DECLARATION OF GENERATIVE AI AND AI-ASSISTED TECHNOLOGIES IN THE WRITING PROCESS

Large language models were used to polish the manuscript. The authors have thoroughly reviewed and edited all content and take full responsibility for the published work.

SUPPLEMENTAL INFORMATION

Supplemental information can be found online at https://doi.org/10.1016/j.patter.2026.101564.

Received: November 28, 2025
Revised: February 25, 2026
Accepted: April 21, 2026
Published: May 19, 2026

REFERENCES

1. Ilievski, F., Hammer, B., van Harmelen, F., Paassen, B., Saralajew, S., Schmid, U., Biehl, M., Bolognesi, M., Dong, X.L., Gashteovski, K., et al. (2025). Aligning generalization between humans and machines. Nat. Mach. Intell. *7*, 1378–1389. https://doi.org/10.1038/s42256-025-01109-4.
2. Wang, L., Zhang, X., Li, Q., Zhang, M., Su, H., Zhu, J., and Zhong, Y. (2023). Incorporating neuro-inspired adaptability for continual learning in artificial intelligence. Nat. Mach. Intell. *5*, 1356–1368.
3. Zador, A., Escola, S., Richards, B., Ölveczky, B., Bengio, Y., Boahen, K., Botvinick, M., Chklovskii, D., Churchland, A., Clopath, C., et al. (2023). Catalyzing next-generation artificial intelligence through neuroai. Nat. Commun. *14*, 1597.
4. Wang, L., and Li, Q. (2025). Convergent multi-modular architecture for adaptive learning in drosophila and artificial intelligence. iScience *28*, 113799.
5. Ebbinghaus, H. (2013). Memory: A contribution to experimental psychology. Ann. Neurosci. *20*, 155–156.
6. Beck, C.D., Schroeder, B., and Davis, R.L. (2000). Learning performance of normal and mutant drosophila after repeated conditioning trials with discrete stimuli. J. Neurosci. *20*, 2944–2953.
7. Pagani, M.R., Oishi, K., Gelb, B.D., and Zhong, Y. (2009). The phosphatase shp2 regulates the spacing effect for long-term memory induction. Cell *139*, 186–198.
8. Menzel, R., Manz, G., Menzel, R., and Greggers, U. (2001). Massed and spaced learning in honeybees: the role of cs, us, the intertrial interval, and the test interval. Learn. Mem. *8*, 198–208.
9. Anderson, M.J., Jablonski, S.A., and Klimas, D.B. (2008). Spaced initial stimulus familiarization enhances novelty preference in long-evans rats. Behav. Processes *78*, 481–486.
10. Bello-Medina, P.C., Sánchez-Carrasco, L., González-Ornelas, N.R., Jeffery, K.J., and Ramírez-Amaya, V. (2013). Differential effects of spaced vs. massed training in long-term object-identity and object-location recognition memory. Behav. Brain Res. *250*, 102–113.
11. Medin, D.L. (1974). The comparative study of memory. J. Hum. Evol. *3*, 455–463.
12. Robbins, D., and Bush, C.T. (1973). Memory in great apes. J. Exp. Psychol. *97*, 344–348.
13. Kukushkin, N.V., and Carew, T.J. (2017). Memory takes time. Neuron *95*, 259–279.
14. Donovan, J.J., and Radosevich, D.J. (1999). A meta-analytic review of the distribution of practice effect: Now you see it, now you don't. J. Appl. Psychol. *84*, 795–805.
15. Shea, C.H., Lai, Q., Black, C., and Park, J.H. (2000). Spacing practice sessions across days benefits the learning of motor skills. Hum. Mov. Sci. *19*, 737–760.
16. Gluckman, M., Vlach, H.A., and Sandhofer, C.M. (2014). Spacing simultaneously promotes multiple forms of learning in children's science curriculum. Appl. Cogn. Psychol. *28*, 266–273.
17. Roediger, H.L., and Byrne, J. (2008). Learning and memory: A comprehensive reference (vol. 2). In Cognitive Psychology of Memory.
18. Sobel, H.S., Cepeda, N.J., and Kapler, I.V. (2011). Spacing effects in real-world classroom vocabulary learning. Appl. Cogn. Psychol. *25*, 763–767.
19. Vlach, H.A. (2014). The spacing effect in children's generalization of knowledge: Allowing children time to forget promotes their ability to learn. Child Dev. Perspect. *8*, 163–168.
20. Smolen, P., Zhang, Y., and Byrne, J.H. (2016). The right time to learn: mechanisms and optimization of spaced learning. Nat. Rev. Neurosci. *17*, 77–88.
21. Solso, R.L. (2024). Theories in Cognitive Psychology: The Loyola Symposium (Taylor & Francis).
22. Toppino, T.C. (1991). The spacing effect in young children's free recall: Support for automatic-process explanations. Mem. Cognit. *19*, 159–167.
23. Philips, G.T., Ye, X., Kopec, A.M., and Carew, T.J. (2013). Mapk establishes a molecular context that defines effective training patterns for long-term memory formation. J. Neurosci. *33*, 7565–7573.
24. Hintzman, D.L., Summers, J.J., and Block, R.A. (1975). Spacing judgments as an index of study-phase retrieval. J. Exp. Psychol. Hum. Learn. Mem. *1*, 31–40.
25. Tzeng, O.J., and Cotton, B. (1980). A study-phase retrieval model of temporal coding. J. Exp. Psychol. Hum. Learn. Mem. *6*, 705–716.

26. Benjamin, A.S., and Tullis, J. (2010). What makes distributed practice effective? Cogn. Psychol. *61*, 228–247.
27. Melton, A.W. (1970). The situation with respect to the spacing of repetitions and memory. J. Verb. Learn. Verb. Behav. *9*, 596–606.
28. Madigan, S.A. (1969). Intraserial repetition and coding processes in free recall. J. Verb. Learn. Verb. Behav. *8*, 828–835.
29. Kukushkin, N.V., Carney, R.E., Tabassum, T., and Carew, T.J. (2024). The massed-spaced learning effect in non-neural human cells. Nat. Commun. *15*, 9635.
30. Yang, Y., Lv, H., and Chen, N. (2023). A survey on ensemble learning under the era of deep learning. Artif. Intell. Rev. *56*, 5545–5589.
31. Ganaie, M.A., Hu, M., Malik, A.K., Tanveer, M., and Suganthan, P.N. (2022). Ensemble deep learning: A review. Eng. Appl. Artif. Intell. *115*, 105151.
32. Wang, L., Zhang, X., Li, Q., Zhu, J., and Zhong, Y. (2022). Coscl: Cooperation of small continual learners is stronger than a big one. In European Conference on Computer Vision (Springer), pp. 254–271.
33. Laine, S., and Aila, T. (2016). Temporal ensembling for semi-supervised learning. Preprint at arXiv. https://doi.org/10.48550/arXiv.1610.02242.
34. Hao, L., Healey, C.G., and Bass, S.A. (2016). Effective visualization of temporal ensembles. IEEE Trans. Vis. Comput. Graph. *22*, 787–796.
35. Srivastava, N., Hinton, G., Krizhevsky, A., Sutskever, I., and Salakhutdinov, R. (2014). Dropout: a simple way to prevent neural networks from overfitting. J. Mach. Learn. Res. *15*, 1929–1958.
36. Rame, A., Kirchmeyer, M., Rahier, T., Rakotomamonjy, A., Gallinari, P., and Cord, M. (2022). Diverse weight averaging for out-of-distribution generalization. Adv. Neural Inf. Process. Syst. *35*, 10821–10836.
37. Polyak, B.T., and Juditsky, A.B. (1992). Acceleration of stochastic approximation by averaging. SIAM J. Control Optim. *30*, 838–855.
38. Hinton, G., Vinyals, O., and Dean, J. (2015). Distilling the knowledge in a neural network. Preprint at arXiv. https://doi.org/10.48550/arXiv.1503.02531.
39. Chen, D., Mei, J.P., Wang, C., Feng, Y., and Chen, C. (2020). Online knowledge distillation with diverse peers. Proc. AAAI Conf. Artif. Intell. *34*, 3430–3437.
40. Zhang, L., Song, J., Gao, A., Chen, J., Bao, C., and Ma, K. (2019). Be your own teacher: Improve the performance of convolutional neural networks via self distillation. In Proceedings of the IEEE/CVF International Conference on Computer Vision.
41. Mumuni, A., and Mumuni, F. (2022). Data augmentation: A comprehensive survey of modern approaches. Array *16*, 100258.
42. DeVries, T. (2017). Improved regularization of convolutional neural networks with cutout. Preprint at arXiv. https://doi.org/10.48550/arXiv.1708.04552.
43. Krizhevsky, A., Hinton, G. (2009). Learning Multiple Layers of Features from Tiny Images.
44. Allen-Zhu, Z., and Li, Y. (2023). Towards understanding ensemble, knowledge distillation and self-distillation in deep learning. In International Conference on Learning Representations.
45. do Santos, C.F.G., Colombo, D., Roder, M., and Papa, J.P. (2021). Maxdropout: deep neural network regularization based on maximum output values. In International Conference on Pattern Recognition (IEEE), pp. 2671–2676.
46. Ghiasi, G., Lin, T.Y., and Le, Q.V. (2018). Dropblock: A regularization method for convolutional networks. In Advances in Neural Information Processing Systems, p. 31.
47. Nguyen, K.B., Choi, J., and Yang, J.S. (2022). Checkerboard dropout: A structured dropout with checkerboard pattern for convolutional neural networks. IEEE Access *10*, 76044–76054.
48. Grill, J.B., Strub, F., Altché, F., Tallec, C., Richemond, P., Buchatskaya, E., Doersch, C., Avila Pires, B., Guo, Z., Gheshlaghi Azar, M., et al. (2020). Bootstrap your own latent-a new approach to self-supervised learning. Adv. Neural Inf. Process. Syst. *33*, 21271–21284.
49. Pang, B., Zhang, Y., Li, Y., Cai, J., and Lu, C. (2022). Unsupervised visual representation learning by synchronous momentum grouping. In European Conference on Computer Vision (Springer), pp. 265–282.
50. He, K., Fan, H., Wu, Y., Xie, S., and Girshick, R. (2020). Momentum contrast for unsupervised visual representation learning. In Proceedings of the IEEE/CVF Conference on Computer Vision and Pattern Recognition, pp. 9729–9738.
51. Li, L., and Jin, Z. (2022). Shadow knowledge distillation: Bridging offline and online knowledge transfer. Adv. Neural Inf. Process. Syst. *35*, 635–649.
52. Li, Z., Li, X., Yang, L., Zhao, B., Song, R., Luo, L., Li, J., and Yang, J. (2023). Curriculum temperature for knowledge distillation. Proc. AAAI Conf. Artif. Intell. *37*, 1504–1512.
53. Sun, S., Ren, W., Li, J., Wang, R., and Cao, X. (2024). Logit standardization in knowledge distillation. In Proceedings of the IEEE/CVF Conference on Computer Vision and Pattern Recognition, pp. 15731–15740.
54. Shen, Y., Xu, L., Yang, Y., Li, Y., and Guo, Y. (2022). Self-distillation from the last mini-batch for consistency regularization. In Proceedings of the IEEE/CVF Conference on Computer Vision and Pattern Recognition, pp. 11943–11952.
55. Kim, K., Ji, B., Yoon, D., and Hwang, S. (2021). Self-knowledge distillation with progressive refinement of targets. In Proceedings of the IEEE/CVF International Conference on Computer Vision, pp. 6567–6576.
56. Li, C., Wang, Z., and Qi, H. (2022). Online knowledge distillation by temporal-spatial boosting. In Proceedings of the IEEE/CVF Winter Conference on Applications of Computer Vision, pp. 197–206.
57. Tully, T., and Quinn, W.G. (1985). Classical conditioning and retention in normal and mutant drosophila melanogaster. J. Comp. Physiol. *157*, 263–277.
58. Tully, T., Preat, T., Boynton, S.C., and Del Vecchio, M. (1994). Genetic dissection of consolidated memory in drosophila. Cell *79*, 35–47.
59. Barth, J., Dipt, S., Pech, U., Hermann, M., Riemensperger, T., and Fiala, A. (2014). Differential associative training enhances olfactory acuity in drosophila melanogaster. J. Neurosci. *34*, 1819–1837.
60. Zhao, B., Sun, J., Zhang, X., Mo, H., Niu, Y., Li, Q., Wang, L., and Zhong, Y. (2019). Long-term memory is formed immediately without the need for protein synthesis-dependent consolidation in drosophila. Nat. Commun. *10*, 4550.
61. Zhou, H., Xiong, G.J., Jing, L., Song, N.N., Pu, D.L., Tang, X., He, X.B., Xu, F.Q., Huang, J.F., Li, L.J., et al. (2017). The interhemispheric ca1 circuit governs rapid generalisation but not fear memory. Nat. Commun. *8*, 2190.
62. Hong, I., Kim, Y., Jung, H., Kim, C.H., Cho, J.H., and Kaang, B.K. (2026). Protein synthesis blockade prevents fear memory reactivation via inhibition of engram synapse strengthening. Proc. Natl. Acad. Sci. USA *123*, e2510016123.
63. Wang, Y., Yao, Q., Kwok, J.T., and Ni, L.M. (2020). Generalizing from a few examples: A survey on few-shot learning. ACM Comput. Surv. *53*, 1–34.
64. Parnami, A., and Lee, M. (2022). Learning from few examples: A summary of approaches to few-shot learning. Preprint at arXiv. https://doi.org/10.48550/arXiv.2203.04291.
65. Song, Y., Wang, T., Cai, P., Mondal, S.K., and Sahoo, J.P. (2023). A comprehensive survey of few-shot learning: Evolution, applications, challenges, and opportunities. ACM Comput. Surv. *55*, 1–40.
66. Wang, L., Zhang, X., Su, H., and Zhu, J. (2024). A comprehensive survey of continual learning: Theory, method and application. IEEE Trans. Pattern Anal. Mach. Intell. *46*, 5362–5383.
67. Sun, G., Yan, H., Wang, L., Kang, Z., Cui, S., Su, H., Zhu, J., and Zhong, Y. (2026). Mepo: Meta post-refinement for rehearsal-free general continual learning. Preprint at arXiv. https://doi.org/10.48550/arXiv.2602.07940.
68. Yan, H., Sun, G., Zhou, K., Li, Q., Wang, L., and Zhong, Y. (2026). Flyprompt: Brain-inspired random-expanded routing with temporal-ensemble experts for general continual learning. Preprint at arXiv. https://doi.org/10.48550/arXiv.2602.01976.

69. De Lange, M., Aljundi, R., Masana, M., Parisot, S., Jia, X., Leonardis, A., Slabaugh, G., and Tuytelaars, T. (2022). A continual learning survey: Defying forgetting in classification tasks. IEEE Trans. Pattern Anal. Mach. Intell. *44*, 3366–3385.
70. Stutz, D., Hein, M., and Schiele, B. (2019). Disentangling adversarial robustness and generalization. In Proceedings of the IEEE/CVF Conference on Computer Vision and Pattern Recognition, pp. 6976–6987.
71. Bai, T., Luo, J., Zhao, J., Wen, B., and Wang, Q. (2021). Recent advances in adversarial training for adversarial robustness. Preprint at arXiv. https://doi.org/10.48550/arXiv.2102.01356.
72. Russakovsky, O., Deng, J., Su, H., Krause, J., Satheesh, S., Ma, S., Huang, Z., Karpathy, A., Khosla, A., Bernstein, M., et al. (2015). Imagenet large scale visual recognition challenge. Int. J. Comput. Vis. *115*, 211–252.
73. He, K., Zhang, X., Ren, S., and Sun, J. (2016). Deep residual learning for image recognition. In Proceedings of the IEEE Conference on Computer Vision and Pattern Recognition, pp. 770–778.
74. Sun, G., Yan, H., Wang, L., Li, Q., Lei, B., and Zhong, Y. (2025). Right time to learn: Promoting generalization via bio-inspired spacing effect in knowledge distillation. In International Conference on Machine Learning (PMLR), pp. 57790–57807.
75. Zhang, Y., Xiang, T., Hospedales, T.M., and Lu, H. (2018). Deep mutual learning. In Proceedings of the IEEE Conference on Computer Vision and Pattern Recognition, pp. 4320–4328.
76. Zhu, X., and Gong, S. (2018). Knowledge distillation by on-the-fly native ensemble. In Advances in Neural Information Processing Systems, p. 31.
77. Li, Q., Zhang, X., Hu, W., Liang, X., Zhang, F., Wang, L., Liu, Z.J., and Zhong, Y. (2016). Importin-7 mediates memory consolidation through regulation of nuclear translocation of training-activated mapk in drosophila. Proc. Natl. Acad. Sci. USA *113*, 3072–3077.
78. Sun, G. (2026). Code for the article "spacing effect improves generalization in biological and artificial system". Zenodo. https://doi.org/10.5281/zenodo.17959984.

Patterns, Volume *7*

Supplemental information

Spacing effect improves generalization in biological and artificial systems

Guanglong Sun, Ning Huang, Hongwei Yan, Jun Zhou, Qian Li, Bo Lei, Yi Zhong, and Liyuan Wang

SUPPLEMENTAL INFORMATION INDEX

Supplemental Information

Figure S1. Impact of variation strength and space interval in cutout augmentation.

Figure S2. Quantitative assessment of training-test similarity and generalization.

Figure S3. Impact of variation strength and space interval on temporal ensemble strategies.

Figure S4. Additional benefits of combining spaced temporal ensemble strategies.

Figure S5. Periodicity in spacing schedules is critical for enhanced generalization.

Figure S6. Impact of spaced training on learning dynamics and performance ceiling.

Figure S7. Impact of parameter perturbations with Gaussian noise.

Figure S8. *Drosophila* exhibit generalization to a similar odor after training.

Figure S9. Massed training with variable shock intensity has no effect on memory.

Figure S10. Impact of protein synthesis inhibition on memory persistence and generalization.

Table S1. Detailed experimental results across multiple run.

Note S1. Algorithm for spaced version of dropout.

Note S2. Algorithm for spaced version of EMA.

Note S3. Algorithm for spaced version of online KD.

Note S4. Algorithm for spaced version of self KD.

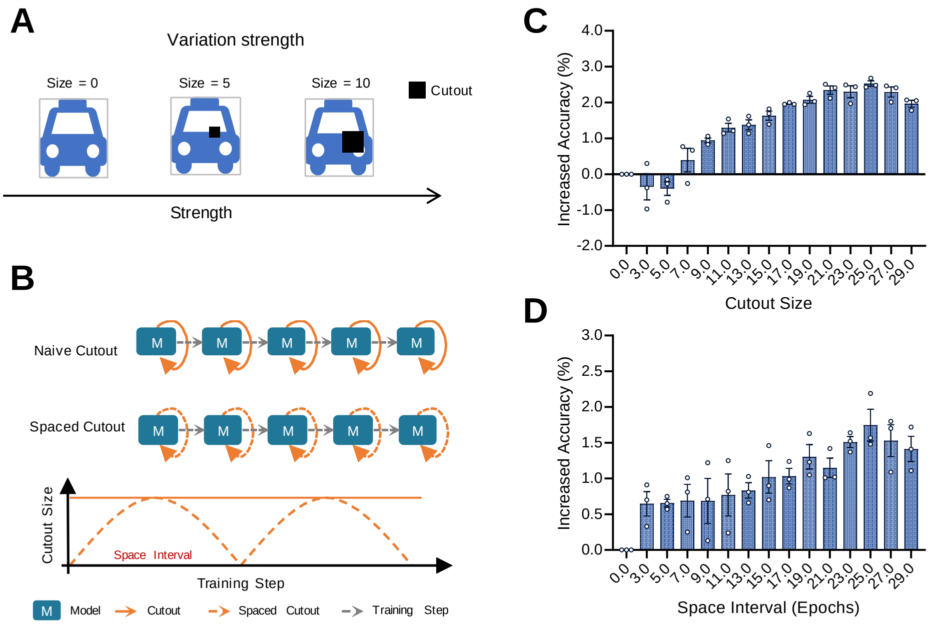

Figure S1: **Impact of variation strength and space interval in cutout augmentation.** (**A**) Variation strength is modulated by the cutout size, which controls the degree of occlusion applied to the input image. (**B**) Spaced interval determines how frequently the cutout size is altered across training epochs. (**C**) Performance gains of ResNet-18 on CIFAR-10 exhibit an inverted U-shaped trend with respect to cutout size. (**D**) Periodically varying cutout size with different spaced intervals also produces an inverted U-shaped curve. All results are averaged over three runs with different random seeds. Data are presented as mean ± SEM.

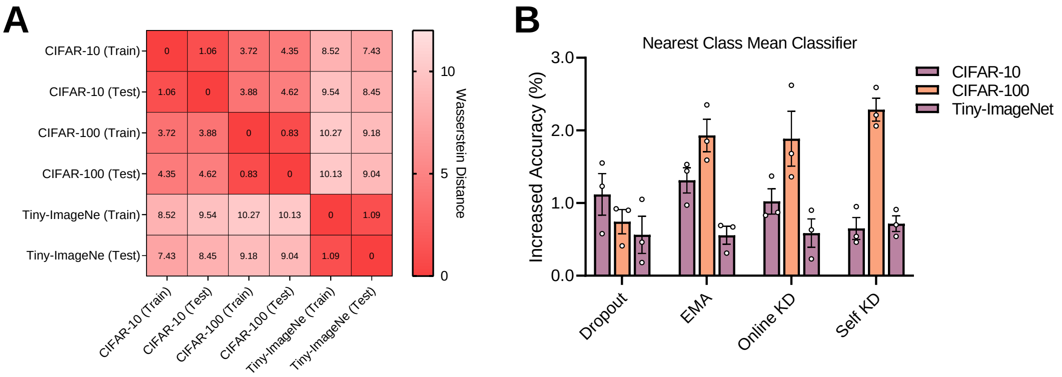

Figure S2: **Quantitative assessment of training-test similarity and generalization.** (**A**) Average data differences under the Wasserstein distance metric. The heatmap displays pairwise distances between 5,000 randomly selected samples from the training and test splits of each dataset. (**B**) Generalization of cross-dataset representations on ResNet-18. The learned representations from the training set of CIFAR-100 are evaluated with a nearest class mean (NCM) classifier on the test sets of CIFAR-10, CIFAR-100, and Tiny-ImageNet. We report the increased accuracy (%) achieved by the Spaced strategies relative to the Naive baselines.

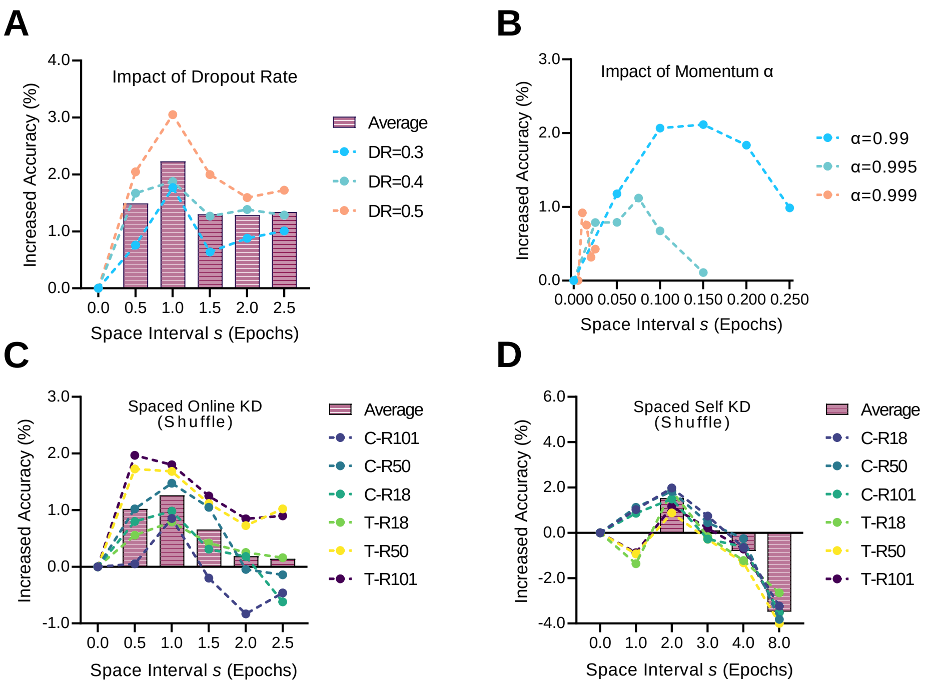

Figure S3: **Impact of variation strength and space interval on temporal ensemble strategies.** (**A**) Performance gains under different dropout rates ($p_{\max} \in \{0.3, 0.4, 0.5\}$) and spacing intervals using the spaced version of dropout. (**B**) Performance gains under different momentum coefficients ($\alpha \in \{0.99, 0.995, 0.999\}$) and spacing intervals using the spaced version of EMA. (**C**) Performance gains when teacher and student models receive shuffled training data at spaced intervals using spaced version of online KD. (**D**) Performance gains when teacher and student models receive shuffled training data at spaced intervals using spaced version of self KD. All results are averaged over three runs with different random seeds. Data are presented as mean ± SEM. See Supplementary Table S1 for the original results of baselines and spaced variants.

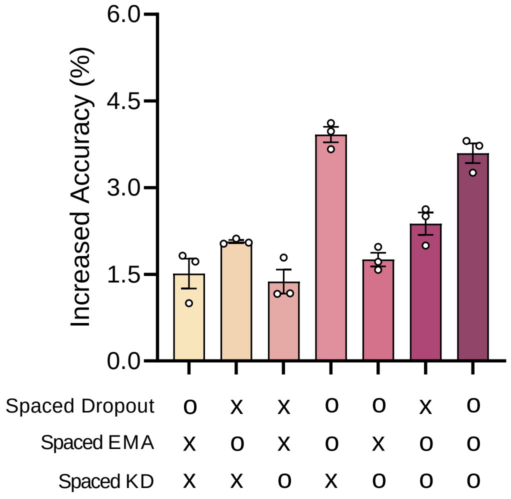

Figure S4: **Additional benefits of combining spaced temporal ensemble strategies.** We evaluate the test accuracy when combining spaced versions of dropout, EMA, and KD with ResNet-18 on CIFAR-100. "○" indicates the strategy is enabled, while "×" indicates it is disabled. To mitigate potential selection bias, the specific spacing intervals applied for each experiment are explicitly reported in the relevant figures and Supplementary Table S1. Data are presented as mean ± SEM over three independent runs.

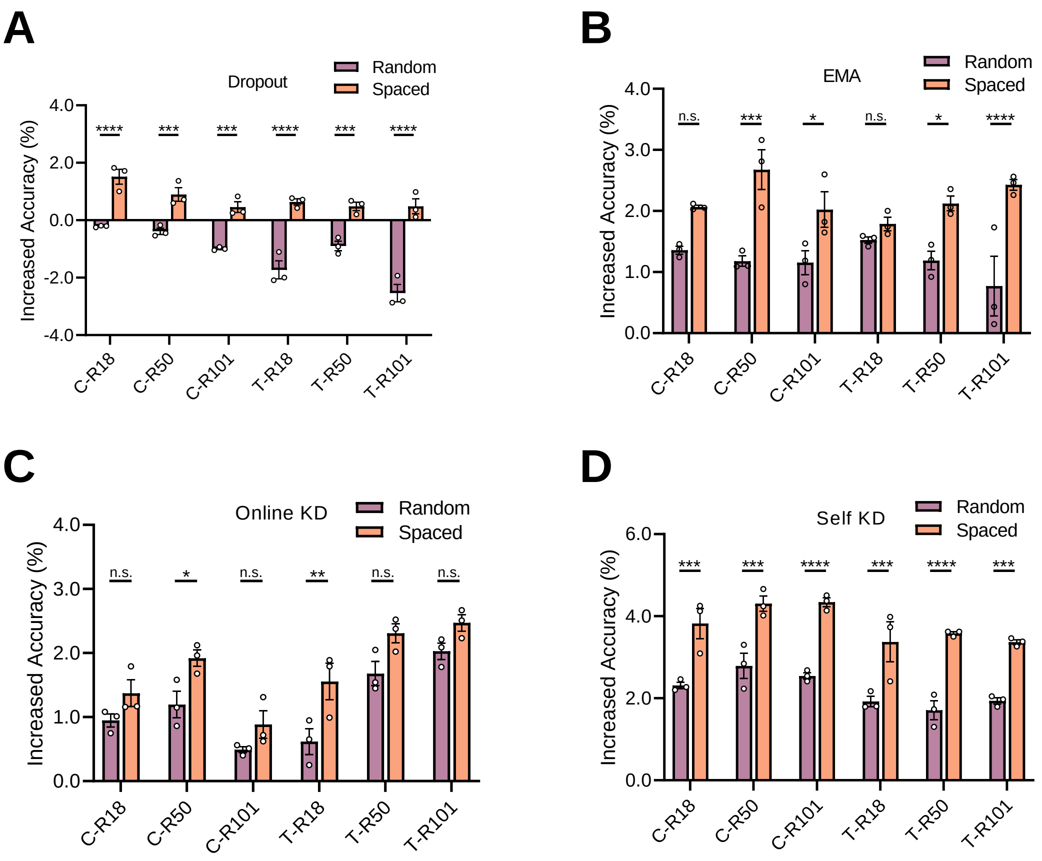

Figure S5: **Periodicity in spacing schedules is critical for enhanced generalization.** We compare the standard "Spaced" strategies (periodic intervals) against "Random" variants where the variation schedule was shuffled (irregular intervals) while maintaining the same average variation frequency. (**A**) Performance comparison for dropout. (**B**) Performance comparison for EMA. (**C**) Performance comparison for online KD. (**D**) Performance comparison for self KD. C, CIFAR-100. T, Tiny-ImageNet. R18, ResNet-18. R50, ResNet-50. R101, ResNet-101. All results are averaged over three runs with different random seeds. Data are presented as mean ± SEM. Statistical significance is determined by one-way ANOVA with Dunnett's multiple comparisons test. *$p \leq 0.05$, **$p \leq 0.01$, ****$p \leq 0.0001$, n.s., not significant.

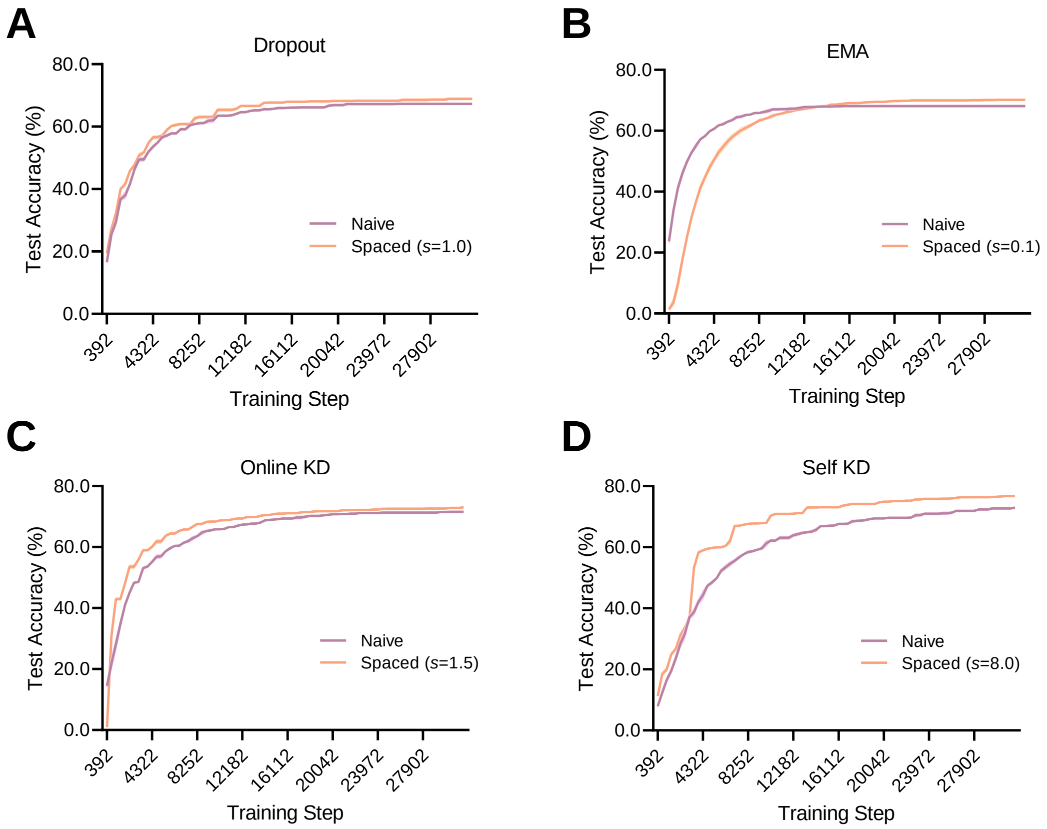

Figure S6: **Impact of spaced training on learning dynamics and performance ceiling.** We compare the test accuracy trajectories of standard "Naive" baselines versus their "Spaced" counterparts over training steps. (**A**) Training curve for dropout. (**B**) Training curve for EMA. (**C**) Training curve for online KD. (**D**) Training curve for self KD. All experiments use ResNet-18 on CIFAR-100. Data are averaged over three independent runs.

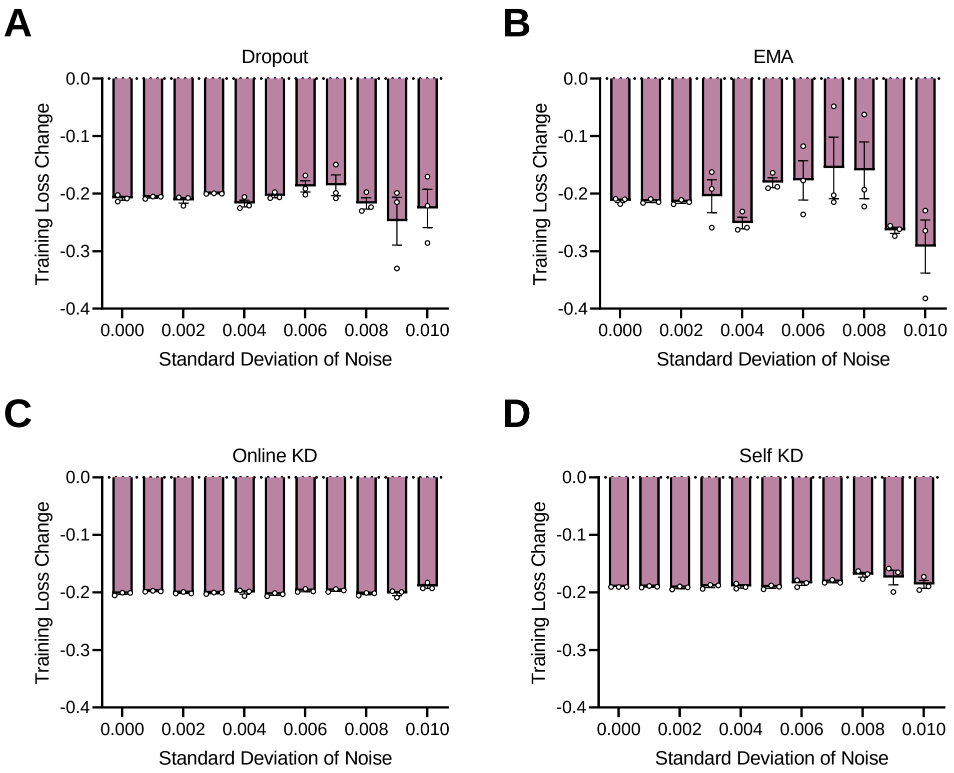

Figure S7: **Impact of parameter perturbations with Gaussian noise.** The bar display the training loss differences ($\Delta\mathcal{L} = \mathcal{L}_{\text{Spaced}} - \mathcal{L}_{\text{Naive}}$) between the standard "Naive" baselines and our "Spaced" counterparts. A negative value indicates that the Spaced variant experiences a smaller increase in training loss compared to the Naive baseline, suggesting a more robust parameterized solution. (**A**) Training loss differences for dropout. (**B**) Training loss differences for EMA. (**C**) Training loss differences for online KD. (**D**) Training loss differences for self KD. All experiments use ResNet-18 on CIFAR-100. Data are averaged over three independent runs.

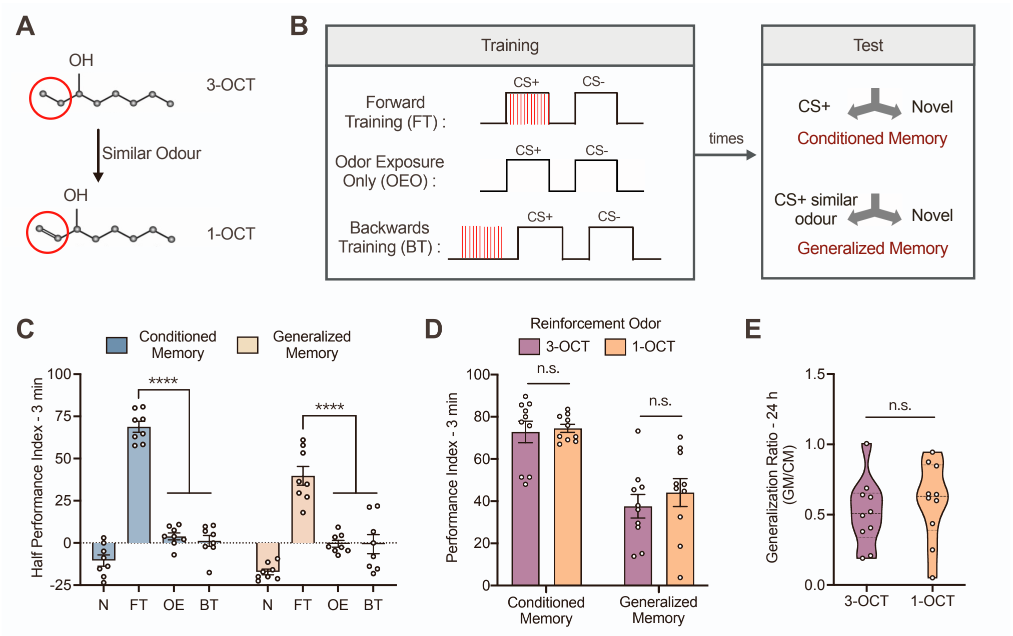

Figure S8: ***Drosophila* exhibit generalization to a similar odor after training.** (**A**) 3-OCT and 1-OCT are chemically similar odors. (**B**) Experimental paradigm for generalization training. Different groups of flies underwent forward training (FT), odor exposure only (OEO), or backwards training (BT). Conditioned and generalized memory are tested at indicated time points by presenting the CS+ odor (3-OCT) or a structurally similar odor (1-OCT), respectively, against a novel odor (MCH). (**C**) Both conditioned and generalized memory in the FT group are significantly higher than in control groups at 3 min. n=8. (**D**) Altering the reinforcement odor does not affect conditioned or generalized memory at 3 min. n=10. (**E**) Altering the reinforcement odor does not affect the generalization ratio at 3 min. n=10. Data are presented as mean ± SEM. Statistical significance is determined by two-way ANOVA with Sidak's multiple comparisons test or unpaired t test. ****p≤0.0001, n.s., not significant.

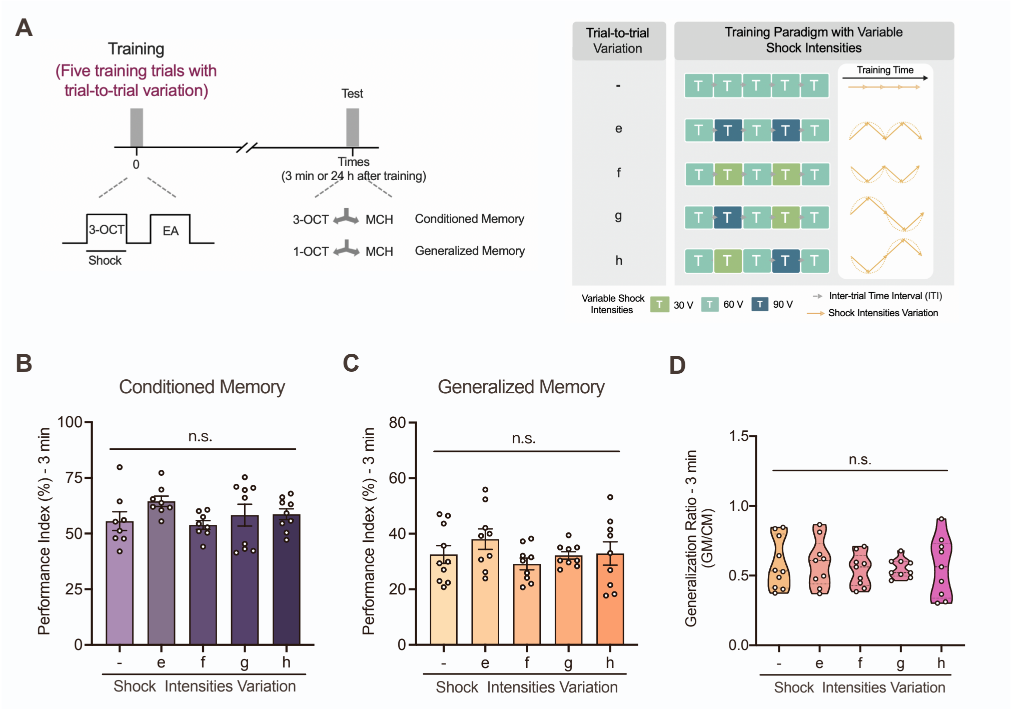

Figure S9: **Massed training with variable shock intensity has no effect on memory.** (**A**) Experimental paradigm for massed training with variable shock input. The control group (-) receives five trials with constant shock intensity, while experimental groups (e-h) receive trials with varying intensities to introduce encoding variability. (**B**-**D**) Conditioned memory (**B**, n=8-9), generalized memory (**C**, n=9-10), and generalization ratio (**D**, n=9-10) at 3 min are all no significant difference between control and experimental groups (e-h). Data are presented as mean ± SEM. Statistical significance is determined by one-way ANOVA with Dunnett's multiple comparisons test. n.s., not significant.

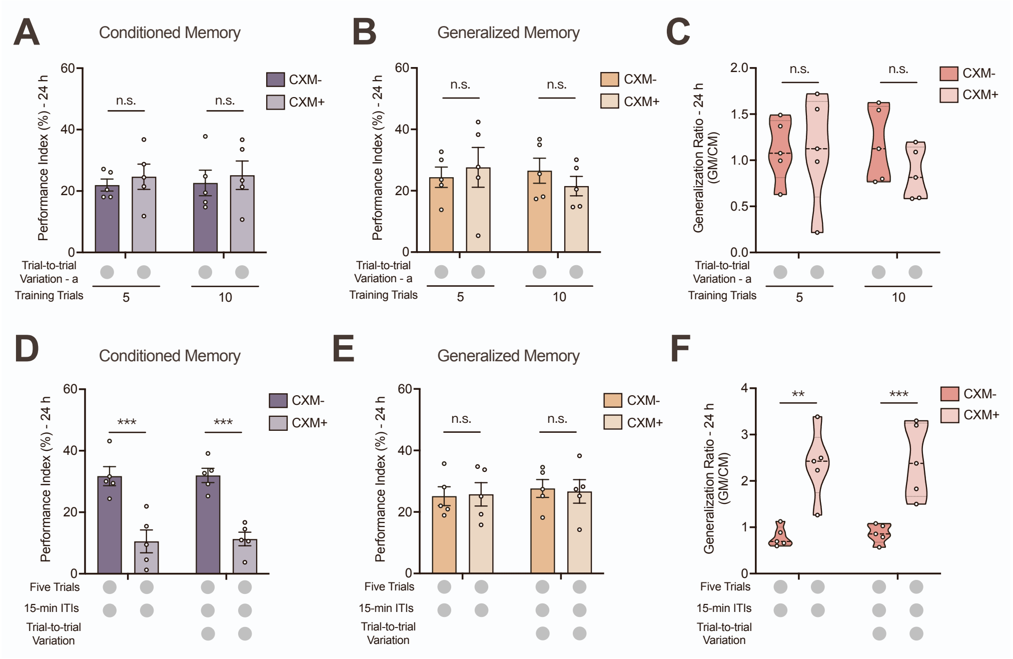

Figure S10: **Impact of protein synthesis inhibition on memory persistence and generalization.** (**A–C**) Trial-to-trial variation enhances generalization independently of protein synthesis during massed training. Conditioned memory (**A**, n=5), generalized memory (**B**, n=5), and generalization ratio (**C**, n=5) measured 24 h after 5 or 10 massed trials with trial-to-trial variation. No significant differences were observed between Control and CXM-treated groups. (**D–F**) Mechanistic dissociation between conditioned memory and generalization enhancement in spaced training. (**D**) CXM treatment significantly abolishes 24 h conditioned memory following spaced training compared to controls. n=5. (**E**) Generalized memory at 24 h remains intact in the CXM-treated group, showing no significant difference from controls. n=5. (**F**) The generalization ratio is significantly elevated in CXM-treated groups. n=5.

Table S1: **Detailed experimental results across multiple runs.** This table reports the absolute test accuracy (%) for three independent runs with different random seeds. We compare the "Naive" baselines against our proposed "Spaced" variants across four temporal ensemble strategies: Dropout (Fig. 2C, $s = 1.0$), EMA (Fig. 2G, $s = 0.1$), Online KD (Fig. 3C, $s = 1.5$), and Self KD (Fig. 3G, $s = 12.0$). Experiments were conducted on CIFAR-100 and Tiny-ImageNet datasets using ResNet-18, ResNet-50, and ResNet-101 backbones. The "Spaced" variants consistently achieve higher test accuracy compared to the naive baselines across all trials and network architectures.

**Method**	**Dataset / Model**	**Naive**			**Spaced (Ours)**		
		Run 1	Run 2	Run 3	Run 1	Run 2	Run 3
Dropout	CIFAR-100 / ResNet-18	67.08	67.46	67.31	68.80	68.46	69.13
	CIFAR-100 / ResNet-50	69.16	68.93	69.31	70.53	69.65	69.91
	CIFAR-100 / ResNet-101	68.97	68.96	69.00	69.22	69.25	69.83
	Tiny-ImageNet / ResNet-18	54.58	54.54	55.26	55.35	55.26	55.68
	Tiny-ImageNet / ResNet-50	57.68	56.85	56.61	58.25	57.05	57.29
	Tiny-ImageNet / ResNet-101	57.74	57.51	57.89	57.85	57.87	58.87
EMA	CIFAR-100 / ResNet-18	68.32	68.17	67.87	70.35	70.29	69.92
	CIFAR-100 / ResNet-50	68.81	68.72	69.12	71.97	71.53	71.18
	CIFAR-100 / ResNet-101	67.62	67.44	67.53	70.22	69.26	69.18
	Tiny-ImageNet / ResNet-18	61.63	61.74	61.56	63.63	63.48	63.18
	Tiny-ImageNet / ResNet-50	64.10	64.49	64.30	66.46	66.55	66.25
	Tiny-ImageNet / ResNet-101	63.95	64.13	64.10	66.21	66.69	66.56
Online KD	CIFAR-100 / ResNet-18	70.97	70.99	71.20	72.76	72.16	72.36
	CIFAR-100 / ResNet-50	71.77	71.72	72.05	73.89	73.68	73.73
	CIFAR-100 / ResNet-101	71.70	72.35	72.03	73.01	73.07	72.65
	Tiny-ImageNet / ResNet-18	59.23	59.50	58.85	61.02	60.49	60.74
	Tiny-ImageNet / ResNet-50	61.11	61.23	60.64	63.49	63.25	63.16
	Tiny-ImageNet / ResNet-101	61.52	61.27	61.09	63.75	63.78	63.76
Self KD	CIFAR-100 / ResNet-18	72.98	72.80	74.09	77.10	77.05	77.18
	CIFAR-100 / ResNet-50	75.60	75.20	75.73	79.84	79.86	79.75
	CIFAR-100 / ResNet-101	76.34	76.14	75.93	80.48	80.51	80.44
	Tiny-ImageNet / ResNet-18	61.51	60.62	61.10	63.92	64.35	65.08
	Tiny-ImageNet / ResNet-50	63.79	63.48	63.58	66.77	67.08	67.08
	Tiny-ImageNet / ResNet-101	62.99	63.90	63.47	67.49	67.49	67.09

Note S1: Algorithm for spaced version of dropout.

Algorithm 1 Spaced version of dropout

Require: model f_θ, dataset $\mathcal{D}_{\text{train}}$, max dropout rate $p_{\max} \in [0, 1]$, epoch number E, space interval s

Ensure: train f_θ with periodically varying dropout rates

for $1 \le e \le E$ **do**
    Compute dropout rate $p_e \leftarrow p_{\max} \cdot \sin\left(\frac{2\pi e}{s}\right)$
    for $(x_i, y_i) \in \mathcal{D}_{\text{train}}$ **do**
        Sample dropout mask $r \sim \text{Bernoulli}(p_e)$
        Forward pass: $h \leftarrow r \odot f_\theta(x_i)$
        Update $\theta \leftarrow \theta - \nabla_\theta \mathcal{L}_{\text{task}}(h, y_i)$
    end for
end for

Note S2: Algorithm for spaced version of EMA.

Algorithm 2 Spaced version of EMA

Require: model f_θ, dataset $\mathcal{D}_{\text{train}}$, momentum α, epoch number E, space interval s

Ensure: train f_θ with momentum updates every s steps

Initialize momentum model $\theta_m \leftarrow \theta$
Initialize step counter $c \leftarrow 0$
for $1 \le e \le E$ **do**
    for $(x_i, y_i) \in \mathcal{D}_{\text{train}}$ **do**
        Update $\theta \leftarrow \theta - \nabla_\theta \mathcal{L}_{\text{task}}(f_\theta(x_i), y_i)$
        $c \leftarrow c + 1$
        if $c \bmod s = 0$ **then**
            Update momentum model: $\theta_m \leftarrow \alpha\theta_m + (1 - \alpha)\theta$
        end if
    end for
end for

Note S3: Algorithm for spaced version of online KD.

Algorithm 3 Spaced version of online KD

Require: student f_θ, teacher g_ϕ, dataset $\mathcal{D}_{\text{train}}$, distillation loss coefficient λ, epoch number E, space interval s

Ensure: train student model using spaced online KD

```
Initialize data index set: R ← ∅
for 1 ≤ e ≤ E do
    for (x_i, y_i) ∈ D_train do
        R ← R ∪ {i}
        Update teacher φ ← φ − ∇_φ L_task(g_φ(x_i), y_i)
        if |R| == s then
            for j ∈ R do
                Retrieve (x_j, y_j) from D_train
                Update student θ ← θ − ∇_θ [λ L_KD(f_θ(x_j), g_φ(x_j)) + (1 − λ) L_task(f_θ(x_j), y_j)]
            end for
            Clear index set: R ← ∅
        end if
    end for
end for
```

Note S4: Algorithm for spaced version of self KD.

Algorithm 4 Spaced version of self KD

Require: model $f_\theta = f_{\theta_1} \circ \cdots \circ f_{\theta_m}$ with m blocks, dataset $\mathcal{D}_{\text{train}}$, distillation loss coefficient λ, epoch number E, space interval s

Ensure: train f_θ using spaced self KD

Initialize data index set: $\mathcal{R} \leftarrow \emptyset$
for $1 \le e \le E$ **do**
  for $(x_i, y_i) \in \mathcal{D}_{\text{train}}$ **do**
    $\mathcal{R} \leftarrow \mathcal{R} \cup \{i\}$
    Update $\theta \leftarrow \theta - \nabla_\theta \mathcal{L}_{\text{task}}(f_\theta(x_i), y_i)$
    if $|\mathcal{R}| == s$ **then**
      for $j \in \mathcal{R}$ **do**
        Retrieve (x_j, y_j) from $\mathcal{D}_{\text{train}}$
        Initialize $\mathcal{L}' \leftarrow \mathcal{L}_{\text{task}}(f_\theta(x_j), y_j)$
        for $1 \le k < m$ **do**
          $\mathcal{L}' \leftarrow \mathcal{L}' + \lambda \mathcal{L}_{\text{KD}}(f_{\theta_1} \circ \cdots \circ f_{\theta_k}(x_j), f_\theta(x_j))$
        end for
        Update $\theta \leftarrow \theta - \nabla_\theta L'$
      end for
      Clear index set: $\mathcal{R} \leftarrow \emptyset$
    end if
  end for
end for
